# Systematic discovery of DNA-binding tandem repeat proteins

**DOI:** 10.1093/nar/gkae710

**Published:** 2024-08-27

**Authors:** Xiaoxuan Hu, Xuechun Zhang, Wen Sun, Chunhong Liu, Pujuan Deng, Yuanwei Cao, Chenze Zhang, Ning Xu, Tongtong Zhang, Yong E Zhang, Jun-Jie Gogo Liu, Haoyi Wang

**Affiliations:** Key Laboratory of Organ Regeneration and Reconstruction, State Key Laboratory of Stem Cell and Reproductive Biology, Institute of Zoology, Chinese Academy of Sciences, Beijing 100101, China; University of Chinese Academy of Sciences, Beijing 100049, China; Institute for Stem Cell and Regeneration, Chinese Academy of Sciences, Beijing 100101, China; Key Laboratory of Organ Regeneration and Reconstruction, State Key Laboratory of Stem Cell and Reproductive Biology, Institute of Zoology, Chinese Academy of Sciences, Beijing 100101, China; University of Chinese Academy of Sciences, Beijing 100049, China; Institute for Stem Cell and Regeneration, Chinese Academy of Sciences, Beijing 100101, China; Key Laboratory of Organ Regeneration and Reconstruction, State Key Laboratory of Stem Cell and Reproductive Biology, Institute of Zoology, Chinese Academy of Sciences, Beijing 100101, China; Institute for Stem Cell and Regeneration, Chinese Academy of Sciences, Beijing 100101, China; Beijing Institute for Stem Cell and Regenerative Medicine, Beijing 100101, China; Key Laboratory of Organ Regeneration and Reconstruction, State Key Laboratory of Stem Cell and Reproductive Biology, Institute of Zoology, Chinese Academy of Sciences, Beijing 100101, China; University of Chinese Academy of Sciences, Beijing 100049, China; Institute for Stem Cell and Regeneration, Chinese Academy of Sciences, Beijing 100101, China; State Key Laboratory of Membrane Biology, Beijing Frontier Research Center for Biological Structure, School of Life Sciences, Tsinghua University, Beijing 100084, China; Tsinghua-Peking Center for Life Sciences, Tsinghua University, Beijing 100084, China; Key Laboratory of Organ Regeneration and Reconstruction, State Key Laboratory of Stem Cell and Reproductive Biology, Institute of Zoology, Chinese Academy of Sciences, Beijing 100101, China; University of Chinese Academy of Sciences, Beijing 100049, China; Institute for Stem Cell and Regeneration, Chinese Academy of Sciences, Beijing 100101, China; National Key Laboratory of Efficacy and Mechanism on Chinese Medicine for Metabolic Diseases, Beijing University of Chinese Medicine, Beijing 100029, China; Key Laboratory of Organ Regeneration and Reconstruction, State Key Laboratory of Stem Cell and Reproductive Biology, Institute of Zoology, Chinese Academy of Sciences, Beijing 100101, China; University of Chinese Academy of Sciences, Beijing 100049, China; Institute for Stem Cell and Regeneration, Chinese Academy of Sciences, Beijing 100101, China; Key Laboratory of Organ Regeneration and Reconstruction, State Key Laboratory of Stem Cell and Reproductive Biology, Institute of Zoology, Chinese Academy of Sciences, Beijing 100101, China; University of Chinese Academy of Sciences, Beijing 100049, China; Institute for Stem Cell and Regeneration, Chinese Academy of Sciences, Beijing 100101, China; University of Chinese Academy of Sciences, Beijing 100049, China; Key Laboratory of Zoological Systematics and Evolution, Institute of Zoology, Chinese Academy of Sciences, Beijing 100101, China; State Key Laboratory of Membrane Biology, Beijing Frontier Research Center for Biological Structure, School of Life Sciences, Tsinghua University, Beijing 100084, China; Tsinghua-Peking Center for Life Sciences, Tsinghua University, Beijing 100084, China; Key Laboratory of Organ Regeneration and Reconstruction, State Key Laboratory of Stem Cell and Reproductive Biology, Institute of Zoology, Chinese Academy of Sciences, Beijing 100101, China; University of Chinese Academy of Sciences, Beijing 100049, China; Institute for Stem Cell and Regeneration, Chinese Academy of Sciences, Beijing 100101, China; Beijing Institute for Stem Cell and Regenerative Medicine, Beijing 100101, China

## Abstract

Tandem repeat proteins (TRPs) are widely distributed and bind to a wide variety of ligands. DNA-binding TRPs such as zinc finger (ZNF) and transcription activator-like effector (TALE) play important roles in biology and biotechnology. In this study, we first conducted an extensive analysis of TRPs in public databases, and found that the enormous diversity of TRPs is largely unexplored. We then focused our efforts on identifying novel TRPs possessing DNA-binding capabilities. We established a protein language model for DNA-binding protein prediction (PLM-DBPPred), and predicted a large number of DNA-binding TRPs. A subset was then selected for experimental screening, leading to the identification of 11 novel DNA-binding TRPs, with six showing sequence specificity. Notably, members of the STAR (Short TALE-like Repeat proteins) family can be programmed to target specific 9 bp DNA sequences with high affinity. Leveraging this property, we generated artificial transcription factors using reprogrammed STAR proteins and achieved targeted activation of endogenous gene sets. Furthermore, the members of novel families such as MOON (Marine Organism-Originated DNA binding protein) and pTERF (prokaryotic mTERF-like protein) exhibit unique features and distinct DNA-binding characteristics, revealing interesting biological clues. Our study expands the diversity of DNA-binding TRPs, and demonstrates that a systematic approach greatly enhances the discovery of new biological insights and tools.

## Graphical Abstract

**Graphical Abstract:**
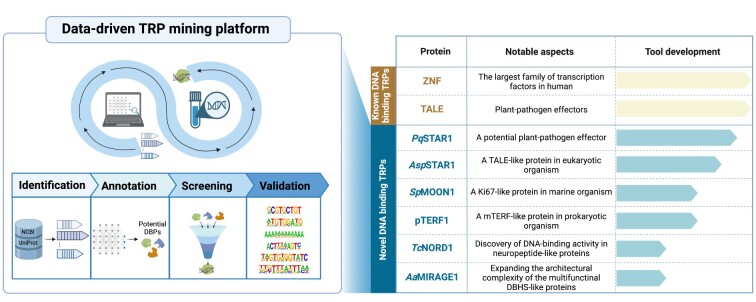

## Introduction

Protein repeats can be classified as tandem repeats (TRs) or non-tandem repeats (NTRs). The units of TRs are continuously arranged in the repeat sequence, whereas the units in NTRs are interspersed (1). TR-containing proteins (TRPs) consist of multiple consecutive units of perfect or imperfect repetitions of a sequence motif, are highly prevalent across all domains of life (2), and exhibit remarkable diversity. Furthermore, TRPs play crucial roles in fundamental biological functions such as ligand binding and transcriptional regulation and are associated with virulence and infectious diseases (3,4).

The repetitive nature of TRPs is often utilized to achieve reprogrammability for recognizing different substrates (5). tetratricopeptide (TPR) (6), WD40 (4), ankyrin (ANK) (7) and leucine-rich (LRR) repeats (8) engage in protein-protein interactions. Sequence variations within these repeats, along with their partner domains, contribute to diverse biological functions. For example, nucleotide-binding site leucine-rich repeat (NBS-LRR) proteins are important for disease resistance in plants, wherein the LRR repeat is involved in determining ligand recognition specificity (9). In addition, the WD40 domain of F-box proteins plays a pivotal role in the recruitment of diverse substrates in the Skp1-Cullin1-F-box (SCF) ubiquitin ligase complex (4).

In addition to protein-binding, TRPs participate in nucleic acid recognition, often in a sequence-specific manner. For example, Pumilio and FBF homology (PUF) and pentatricopeptide repeat (PPR) proteins exhibit modular and sequence-specific RNA binding capability, a feature that has been harnessed to engineer customized RNA-targeting scaffolds (10,11). Furthermore, zinc finger proteins (ZNFs) are encoded by approximately 3% of the human proteome and play key roles in human biology and health (12,13). ZNFs are composed of multiple ‘finger’ units, where each finger forms a ββα configuration in which individual amino acids in the α-helix interact with specific nucleotide bases (12,13). In addition, transcriptional activator-like effectors (TALEs), derived from the plant pathogen *Xanthomonas*, play important roles in pathogen virulence by regulating the transcription of host genes (14). TALEs consist of repeated units of 34 amino acids, with two variable residues determining base pair specificity (15). Following the same logic, ZNFs and TALEs can be reprogrammed to target a predetermined DNA sequence by combining repeat units with varied specificity-determining residues (16,17). Through fusion with nucleases, transcription regulators, or other effectors, these TR-derived site-specific DNA binding systems greatly contribute to the genome and epigenome editing toolbox (17,18).

Among the large number of TRPs that exist in nature, only a very small portion have been experimentally studied, and even fewer have been developed into biotechnological tools. The exponential increase in genomic sequencing data provides raw material for mining novel TRP families. However, very few relevant large-scale studies have been performed, and most of them are limited to comparative or evolutionary analysis of TRPs in plants or mammals, without experimental validation (19–21).

Compared to genome sequencing, the experimental study of novel proteins comes at a much higher cost and slower pace. Hence, employing computational methods to infer functions from sequences can greatly enhance the efficiency of protein functional study. Researchers have recently adapted natural language processing (NLP) techniques for protein analysis, harnessing structural analogies between sentences and protein sequences. By leveraging the wealth of information embedded within protein sequences, precise predictions can be made concerning protein functions, structures, and interactions (22,23). State-of-the-art protein language models (PLMs), such as ProteinBERT (24), ProtTrans (25) and ESM (26), have shown remarkable performance across a diverse range of tasks (27,28). However, there are currently no studies on the integration of different PLMs for predicting functional TRPs.

Considering the importance of TRPs in biology, biotechnology and the large amount of unexplored biodiversity, we set out to establish a platform composed of deep learning-empowered bioinformatic analyses and standardized functional screening and validation pipelines to discover and characterize novel TRPs systematically. We first analyzed and categorized all the TRPs in major public databases and then focused on mining DNA-binding TRPs. In total, we identified 11 novel DNA-binding TRPs, six of which bound to DNA in a sequence-specific manner. Our discovery framework enhances the efficiency of identifying and characterizing novel functional TRs, and the newly discovered DNA-binding TRPs lead to new biological insights and potential novel biotechnological tools.

## Materials and methods

### Computational pipeline for characterizing repeat proteins

Due to continuous updates of the database, our analysis process has been refined on three occasions, specifically in August 2019, August 2021 and August 2022. The synthesized proteins referenced in this article are based on data from the first two updates. All the data analyzed in this article is sourced from the most recent database update in August 2022.

All unique proteins were downloaded from UniRef100 and NCBI-nr database (frozen in August 2022). A three-step pipeline was implemented to characterize proteins with TRs. Firstly, proteins containing periodic repeats were identified by XSTREAM which utilizes the short seed extension method to detect protein TRs (29). Secondly, to classify novel and known TRs, we first collected the well-known TR families (listed in Supplementary Table S1) from literatures and retrieved curated proteins within these families in the UniProtKB/Swiss-Prot database (30–33). Next, we extracted repeat region sequences from these known TR proteins. Subsequently, these sequences were employed as queries for a comprehensive search across all repeat regions of the TRs identified in the initial step. Hits exhibiting at least 30% identity and 70% coverage were designated as putative known TRs and were further validated through domain annotation. Finally, both known and novel TR clusters were obtained using MMseqs2 (34) with the following parameters: -c 0.7 –min-seq-id 0.3 –cov-mode 1 –cluster-mode 1. Considering that repeats can occur in non-integer multiples and their boundaries often do not coincide, we utilized two instances of the consensus sequence for the clustering process.

To investigate the underlying reasons for the significant prevalence of orphan TRs in novel type, we performed the following analysis. Firstly, we analyzed the count of genus-level assemblies through a two-step process: (i) identifying the originating species and their corresponding genus; (ii) using the Entrez Direct E-utilities’ esearch commands to retrieve the count of genus-level assemblies (35). Secondly, we assessed the completeness of genome assemblies to which TRs belong. Specifically, for TRs within each cluster size range, we retrieved the corresponding genome assemblies and then randomly selected 2,000 of them for assessing genome completeness using BUSCO (36).

### Generation of protein identifier

We developed a hierarchical naming scheme for the ‘TRs of interest’ dataset, which incorporates three levels of classification: period, cluster size and unit number (Supplementary Figure S1E). Specifically, (i) all TRs were initially categorized into 21 major groups based on their period length (15, 16, …, 35); (ii) within each period length group, proteins were further sorted by cluster size (C1 to C100); (iii) in cases where two clusters shared identical period lengths and cluster sizes, their ranking was then determined by the median of unit number. Taking the unique identifier ‘TR_15_C1_1’ as an example, TR represents tandem repeat, 15 signifies the period length of 15, C1 indicates ‘Cluster 1’ and 1 represents the TR’s position within its respective cluster, determined by sorting the unit numbers in ascending order (Supplementary Figure S1E).

### Correlation analysis

The correlation analysis in this study was calculated using Pearson correlation coefficients (PCC) and was performed in the RStudio environment (37).

### Evaluation of sequence complexity

Sequence complexity within a repeat was represented by normalized Shannon's entropy score (NSS) (38,39). Consensus sequences of TRs were used for Shannon score calculating. Specifically, the Shannon entropy score is defined as the negative of a sum of the products of amino acid frequencies in a typical repeat sequence (*P_i_*) and binary logarithms of those frequencies (log_2_(*P_i_*)). The calculated Shannon score was then normalized by the length of consensus sequences.


(1) \begin{eqnarray*}&&Normalized\ \textit{Shannon}{\mathrm{\text{'}}}s{\mathrm{\ }}\textit{entropy}{\mathrm{\ }}\textit{score}{\mathrm{\ }}( {{\mathrm{NSS}}} )\nonumber\\ &&\quad= \left(-\sum\limits_{i=1}^{n}{P_ilog}_{2}P_i\right)/log_2(period)\end{eqnarray*}

### Identity cutoff selection

To determine a valid identity threshold for distinguishing different TR families, we collected 100 members of eight well-known TRP families, namely ZNF, ANK, ARM, LRR, PPR, TPR, WD40 and TALE. Inter- and intra-repeats represent repeats between different TRP families and within the same TRP family, respectively. Identity values between inter- and intra-repeats were calculated by using the EMBOSS needle program with default parameters (40). Two copies of consensus sequences were used as input.

### Training datasets generation

A training dataset was constructed to train the proposed predictor based on the UniProtKB/Swiss-Prot database (30). The positive dataset was generated as follows: (i) retrieval of all DNA binding proteins (DBPs) from UniProtKB/Swiss-Prot through a keyword search for ‘DNA binding’; (ii) filtering proteins without a GO term containing ‘DNA binding’; (iii) elimination of sequences that are < 60 amino acids (aa) or > 4000 aa; (iv) elimination of sequences that contain ‘*X*|*x*’ and ‘*J*|*j*’ characters; (v) grouping the remaining sequences that shared a sequence similarity of ≥ 50% by MMseqs2. The non-DNA binding proteins (NDBPs) dataset was generated by the following steps: (i) retrieval of sequences that had a < 25% sequence similarity to any sequences in positive dataset; (ii) filtering proteins with any description that contains ‘DNA binding’; (iii) elimination of sequences that contain ‘*X*|*x*’ and ‘*J*|*j*’ characters; (iv) Grouping the remaining sequences that share a sequence similarity of ≥ 50% by MMseqs2. Totally, 12,989 DBPs and 121,455 NDBPs were generated. To balance the number of DBPs, 12,989 NDBPs were randomly selected for training, denoting as UniSwiss25978 dataset.

### Test datasets generation

An independent test dataset was procured from the Protein Data Bank (PDB) (41), which satisfied the following criteria similar as the training dataset generation, except for the following additions: (i) filtering out sequences that had a ≥ 25% sequence identity to any other sequences in the training datasets utilized by the evaluated tools; (ii) grouping the remaining sequences that shared a sequence identity of ≥ 25% by MMseqs2. A total of 359 representative DBPs and 364 representative NDBPs were generated after fulfilling the filtration criteria. Subsequently, 300 DBPs and NDBPs were randomly chosen, resulting in the PDB600 dataset. The performance of each tool was evaluated using several parameters, including accuracy, specificity, recall, precision, F1 score and area under the curve (AUC). The Receiver operating characteristic (ROC) curves were plotted by using the R ‘pROC’ package (42).

### DBP prediction model construction

The DBP prediction model, PLM-DBPPred, was constructed by integrating the ProteinBERT, ProtTans and ESM pre-trained transformer models (24–26). More specifically, the predicted results from these three models are obtained independently, and the average of the three probabilities is calculated as the final result. The cutoff was set as 0.5.

### ProteinBERT

ProteinBERT is pre-trained in a self-supervised manner using ∼106 million protein sequences from the UniRef90 database, along with their corresponding functional annotations from the gene ontology database. Initially, the model takes protein sequences as input and processes them through a feature encoding step. The resulting embedding vectors are then subjected to attention calculations through a dedicated attention layer. To reduce feature dimensionality, the output of the attention layer is transformed via a fully connected layer, followed by a sigmoid activation function. Furthermore, additional layers of attention and fully connected components are consecutively stacked after the initial one. In addition to employ a two-layer attention for classifier, we also utilized fully connected layers for comparison. The final output of this model is a probability score that signifies whether a given protein exhibits DNA-binding capabilities or not.

In the model training process, all layers of the pre-trained model were initially frozen, with the exception of the attention and fully connected layers, which were trained for up to 10 epochs. Subsequently, all layers were unfrozen and trained for an additional three epochs. To ensure optimal learning, the dynamic learning rate adjustment technique, ReduceLROnPlateau, was employed. The loss was calculated using binary cross-entropy, and an early stopping policy was applied to prevent overfitting. Utilizing the pre-trained model, the entire training procedure was completed on a single GPU, Tesla P100-PCIE-12GB, with the learning rate and batch size set to 0.0001 and 32, respectively.

### ProtTrans

For ProtTrans-based architectures, six models were trained by combining two architectures from ProtTrans (ProtT5-XL-UniRef50 and ProtBERT-BFD) with three different classifiers. The ProtT5-XL-UniRef50 is a variant of the T5 model (43), designed as an encoder-only architecture. It was initially trained on the BFD dataset and then fine-tuned on the UniRef50 dataset. The ProtBERT-BFD is based on the BERT architecture and trained on the BFD dataset. Following the PLMs, three classifiers were used to process the embedding information. The first one is a vanilla MLP, serving as a baseline for classification performance. The second one is the light attention (LA) classifier, which employs a pair of 1D convolution to extract pivotal information (44). The third classifier is biLSTM_TextCNN, which is known for its effectiveness in sentiment classification (45). Finally, probabilities signify whether a given protein exhibits DNA-binding capabilities or not were obtained.

In the model training phase, all layers of the pre-trained ProtT5-XL-UniRef50 model were kept frozen. The classifier was trained for a duration of 10 epochs. The batch size was set to 64. The learning rate was initialized at 0.00005 and optimization was performed using the Adam with a weight decay of 0.001. Similar to the operations in ProteinBERT, the learning rate was dynamically adjusted based on validation set performance using the ReduceLROnPlateau. The entire fine-tuning procedure was completed utilizing a single GPU on Tesla P100-PCIE-12GB.

### ESM

As for ESM-based models, we select the esm2_t30_150M_UR50D and esm2_t33_650M_UR50D model for encoding. After obtaining protein sequence embeddings through the processing phase, these embeddings are subsequently directed to the MLP, LA and biLSTM_TextCNN classifier, following the methodology outlined in ProtTrans.

During the model training process, we performed fine-tuning by unfreezing the embedding norm before layers, embedding norm after layers and the Roberta head for masked language modeling layers within the pre-trained ESM models, in combination with three different classifiers. The training spanned 10 epochs, with the batch size set to 120 and the learning rate initialized at 0.01. Optimization was performed using the AdamW with a weight decay of 0.0001. We also employed the ReduceLROnPlateau to dynamically adjust the learning rate based on validation set results. The entire fine-tuning procedure was completed on 8 Tesla P100-PCIE-12GB GPUs.

### Gene ontology (GO) enrichment analysis of DBPs and NDBPs

The dataset used for DBP GO enrichment were generated similar to the test dataset used for evaluating the DBP prediction tool, with the exception that sequences sharing similarity with any sequences in the training datasets used by the evaluated tools were not removed. PANNZER2 (46) was applied for GO term identification and terms with a PPV > 0.6 were subsequently submitted to clusterProfiler for GO enrichment (47).

### DNA binding domains (DBDs) investigation and protein domain annotation

DNA binding domains (DBDs) were obtained from various sources, including databases such as AnimalTFDB 4.0 (48), PlantTFDB 5.0 (49) and the DNA-binding domain (DBD) database (50). Additionally, a manual keyword search was conducted in the Pfam database for terms such as ‘DNA binding’ and ‘transcription factor’ to ensure comprehensive coverage. Partner domains aside from the DBDs were extracted based on the domain architecture file from the Pfam FTP site. The top 10 enriched partner domains of each DBD were collected and GO terms derived from Pfam2GO were used for conducting GO enrichment (http://www.geneontology.org/external2go/pfam2go). DNA-related domain (DRD) accessions were extracted and manually verified according to the following keywords: DNA binding, RNA binding, nucleic acid binding, transcription factor, nuclease, helicase, deaminase, integrase, ligase, transposase, polymerase, methylase, recombinase. All related accessions were listed in Supplementary Table S5. Proteins annotated with any DNA related domains were added into DRD list.

All repeat proteins were scanned for functional domain entries in the Pfam database (V35.1) by using hmmsearch (51) with the ‘–cut_ga’ option.

### Enrichment analysis of different function annotation

Enrichment analysis of different function was referred to the GO enrichment (52). Take DBP enrichment as example, where A is the count of DBPs in clusterX, B is total number of proteins in clusterX, C is the count of DBPs outside of clusterX, and D is total number of proteins outside of clusterX. The odds ratio was calculated as (A/B)/(C/D) for a specific function annotation. Subsequently, the log_2_ odds ratio (LR) was determined to represent the enrichment score of a particular function for each cluster (52). A larger LR indicates a higher level of enrichment of the function within the cluster compared to the overall sample, and vice versa.

### Protein structure predictions

Protein structure model predictions were conducted by using the ColabFold v1.5.2-patch platform with default parameters (https://colab.research.google.com/github/sokrypton/ColabFold/blob/main/AlphaFold2.ipynb) (53).

### Construction of phylogenetic trees

Repeat region sequences were used to construct the phylogenetic tree for all TRP family, with the exception of MOON and STAR families, which were generated using full length sequences. All phylogenetic trees were constructed by FastTree program (54).

### Transcription activation domain prediction

The transcription activation domain was predicted by ADpred (55).

### Type III secretion signal prediction

The Type III secretion signal (T3SS) was predicted by EffectiveDB software suite (http://effectivedb.org) (56). The cutoff was set as 0.99.

### Structure comparison

Structural comparison was conducted through the computation of the root mean-square deviation (RMSD) matrix, utilizing the R ‘Bio3d’ package (57). The resulting matrix was subsequently visualized by the headmap.2 function in R ‘gplots’ package (58).

### Target gene analysis

Putative target genes of identified DNA-binding TRPs were determined through the following steps: (i) Extracting the promoter sequence (−1000 bp to the TSS site). (ii) Searching the promoter region for enriched motif by using the program FIMO in the MEME Suite. (iii) Extracting the putative target genes from the target hits. (iv) Performing GO annotation for all proteins in genome by PANNZER2 (46). (v) Performing GO enrichment for potential target genes by clusterProfiler (47).

### Taxonomic breadth of TR clusters

For estimation of the taxonomic breadth of each TR cluster, we calculated the last common ancestor (LCA) of their members using Taxonkit v0.13.0 (59). Specifically, we first clustered all identified 4,575,091 TRs by using MMseqs2 (34) with the following parameters: -c 0.7 –min-seq-id 0.3 –cov-mode 1 –cluster-mode 1. Subsequently, the computation of the LCA was executed for all TR cluster containing a minimum of 10 members.

### Protein expression and purification

The pET28a vector in frame with the N-terminal His tag and SUMO tag were used to express and purify protein *in vitro*. Expression constructs for all candidates were synthesized by BGI after codon optimization for *E. coli*. The assembled genes were constructed into a pET28a expression vectors and then transformed into Rosetta *E. coli* cells. For protein expression, 0.1 mM IPTG were added when OD600 reached to 0.6–0.8 and then incubated at 16°C for 18 h. Cell pellets were resuspended in binding buffer (50 mM Tris–HCl, 500 mM NaCl) before lysis by sonication (200 W, 3 s on/3 s off on ice for 10 min). The supernatant was loaded onto a HisTrap HP column (GE Healthcare) after column washing with binding buffer with 5 column volumes. The protein was eluted with binding buffer containing 300 mM imidazole. Molecular sieve chromatography with Superose 6 or Superose 12 HR16/50 was then performed to obtain protein of high purify.

### Bio-layer interferometry (BLI)-based screening

The BLI-based screening method was developed based on the findings of Marklund *et al.* that DNA-binding proteins rapidly associate and dissociate across various sequences but efficiently rebind to the target sequence via searching, leading to macroscopic specific binding (60). Accordingly, the association of a DBP with a random dsDNA library could occur, and the binding signal could be reflected by the response captured by the BLI experiment.

Feasibility testing of BLI-based screening began with comparisons of responses between well-established DBPs and NDBPs. The DBPs included (i) T_AAVS1, a TALE designed to target the *AAVS1* locus (61) and (ii) Zif268, a natural zinc-finger protein (62). The NDBPs included (i) PUM1, the human Pumilio homolog 1 protein (63), (ii) SUMO, a solubility tag (64) and (iii) ULP1, ubiquitin-like-specific protease 1. Two different loading densities were tested (1 or 5 nm). The experimental protocol was set as follows: BLI-based screening was performed on the Octet RED system (Fortebio) at 25°C. The Ni-NTA (NTA) biosensors were dipped in the BLI assay buffer (PBS, 0.02% Tween, pH 7.4) for 10 min before using. The 88-bp dsDNA library with 60-nt randomized regions were annealed and diluted to 100 nM. The dsDNA product was purified by PAGE-gel extraction. Three replicate series were used for each protein. The procedures steps included: baseline for 60 s, loading His-tagged protein, another baseline for 60 s, association for 60 s, dissociation for 30 s and regeneration for 30 s. The response scores were collected and used for further data processing.

### Systematic evolution of ligands by exponential enrichment (SELEX) screening

SELEX assay was performed according to several previous studies (65,66). The dsDNA library contains 20-nt random sequences. For each round of selection, 200 ng purified protein with 3 μL Dynabeads™ His-Tag Isolation and Pulldown beads (Thermo, #10103D) were incubated for 30 min at 25°C. After the unbounded protein removal, 2 μg dsDNA library was added to protein-beads complex and incubated with 100 μL SELEX buffer (50 mM Tris, 150 mM NaCl, 20 mM KCl, 2.5 mM MgCl_2_, 10 μM ZnCl_2_, 0.05% Tween20, 0.01% BSA, 20 μg/ml dIdC). After 1 h incubation at room temperature, unbounded dsDNA was washed away with SELEX buffer five times. Bounded dsDNA was then amplified for the next round selection. After five cycles, recovered DNA fragments were cloned and sequenced.

### Bacteria-one-hybrid (B1H) screening

The plasmids for B1H system were purchased from Addgene (#12609). The 18-nt random sequences library reporter of B1H screening were constructed by T4 ligase with the sticky ends being generated by *Not*I and *EcoR*I. To conduct the B1H assay, a reporter vector with 18-nt random sequences library and a protein expression vector were co-transformed into US0 *E. coli* strain by electroporation. The successful binding events were enriched on plates with 10 mM 3-AT. The enriched cells were scraped from the plates, followed by plasmid extraction and sequencing.

### GFP activation validation

The GFP activation validation system was constructed based on the B1H screening system. Specifically, the *HIS3* marker gene was substituted with the *GFP* reporter gene (Figure 2D). Moreover, the 18-nt random sequence upstream of the promoter was changed to the enriched motif of interest. Subsequently, the binding affinity of tested motifs is indicated by the GFP signal which could be detected by flow cytometry.

### GFP repression validation

The GFP repression system was the modified version of the PAM-SCANR system which was first established to identify functional PAM diversity across CRISPR-Cas systems (67). Specifically, the *lacI* and promoter of *lacI* were deleted and the PAM sequence on reporter plasmid was substituted into the enriched motif of interest (Figure 2D). After the co-transformation of protein expression plasmid and the reporter plasmid, the successful binding of tested motifs with protein can block the expression of GFP, leading to a decreased level of GFP signal detected by flow cytometry.

### Electrophoretic mobility shift assays (EMSA)

For EMSA assay, both FAM labeled or unlabeled probes were generated by annealing oligos. Different protein concentration series were designed for each reaction with the last two lanes added with specific/non-specific unlabeled probes, which set as binding competitors. Binding reactions were incubated at room temperature for 30 min and resolved on a 6% native PAGE gel for 1 h at 80 V. The gels were visualized with a UV transilluminator (Bio-Rad).

### Negative staining sample preparation, data collection and 2D classification average

Individual proteins were purified following the procedures described above. All the complexes were reconstituted by incubating protein and DNA in a 1:10 ratio on ice for 30 minutes in a binding buffer containing 10 mM Tris–HCl (pH 8.0) and 150 mM NaCl.

For the preparation of negative staining samples, all specimens were diluted to a final concentration of 0.5 μM and subjected to negative staining in a 2% (w/v) uranyl acetate solution, following the standard deep-stain protocol on holey-carbon coated EM copper grids covered with a thin layer of continuous carbon (68). The negatively stained specimens were subsequently imaged using a FEI Tecnai-F20 electron microscope operating at an acceleration voltage of 200 kV. Images were captured at a nominal magnification of 50 000×, with a defocus range of 2.5–3.5 μm. These electron micrographs were recorded using a Gatan Ultrascan4000 4k × 4k CCD camera.

Subsequently, the acquired micrographs were processed as negative stain data in CryoSPARC (69). Manual Picker was utilized to select individual particles, and 2D reference-free classification was employed to determine the average particle size.

### BLI for binding kinetics

BLI experiments were performed on the Octet RED system (Fortebio) at 25°C. The streptavidin (SA) sensors were dipped in the assay buffer (PBS, 0.02% Tween, pH7.4) for 10 min before use. Complementary pairs of labelled oligonucleotides (5′ biotin) were annealed in 10 × annealing buffer (Thermo, TECH TIP # 45). The dsDNA product was purified by PAGE-gel extraction. A six-point concentration series were designed for each protein with the last one diluted with PBST buffer, which set as reference. The procedures were set as follows: baseline for 60 s, loading the biotin-conjugated dsDNA for 120 s, another baseline for 120 s, association for 120 s, and dissociation for 120 s. Dissociation (*k*_dis_) and association rate constants (*k*_on_) were determined with the Octet Data Analysis Software, as a result of a global fit considering the entire step times, and assuming a 1:1 binding model.

### Assembly of artificial STAR proteins

The one-step construction of artificial STAR proteins followed the previously reported Golden Gate method, with several refinements (70). Specifically, the repeat module on pHD-1 was replaced with modules derived from *Pq*STAR1, each possessing a unique overhang. Additionally, we implemented modifications to the pFUS_A vector by inserting the N- and C-terminal regions of *Pq*STAR1 at both ends of *LacZ*, respectively. Two internal *BsaI* sites were strategically placed at the 5′ and 3′ termini of *LacZ* to facilitate vector linearization with the enzyme, resulting in the generation of suitable overhangs for the incorporation of the repeat modules.

### Cell lines

Human 293T cells were cultured in DMEM containing 10% foetal bovine serum medium (Invitrogen, Carlsbad, USA) with 1% penicillin/streptomycin (Millipore, TMS-AB2-C). All cells were incubated in a humidified incubator at 37 °C with 5% carbon dioxide.

### Cleavage under targets and tagmentation (CUT&Tag) experimental procedure

Plasmids vectors for protein expression in human 293T cells were constructed with an Ef1a promoter and a 3X FLAG tag at C-terminal. A P2A-GFP sequence following the FLAG tag was used to detect the plasmid transfection efficiency. The 293T cells were seeded into 24-well plates at a density of 1.2 × 10^5^ cells/well. For transfection, 1 μg of plasmid was used with Lipofectamine2000 (Invitrogen). Transfected cells were then cultured at 37°C for another 24 h before conducting the western blot and CUT&Tag assays. CUT&Tag assay was performed with CUT&Tag Assay Kit (TD903, Vazyme Biotech) following the manufacturer's instructions.

### RNA-seq experimental procedure

Plasmids vectors for STAR-based ATFs expression in human 293T cells were constructed with a CMV promoter. A P2A-GFP sequence following the VPR activator domain was used to detect the plasmid transfection efficiency. The 293T cells were seeded into 6-well plates at a density of 8 × 10^5^ cells/well. For transfection, 2.5 μg of plasmid was used with Lipofectamine2000 (Invitrogen). Transfected cells were then cultured at 37°C for another 48 h before RNA extraction. Total RNA was extracted by using TRIzol regent and sequencing was done by the Annoroad Gene Tech. (Beijing) Co., Ltd.

### Sequencing and data processing

SELEX, B1H and CUT&TAG samples were sequenced on the Illumina NovaSeq PE150 platform. RNA-seq samples were sequenced on the MGIDNBSEQ T7 platform. Read trimming and filtering were performed using Trimmomatic version 0.33 (71).

For SELEX and B1H data, after separating samples by barcodes, random DNA library region of each sample was extracted by mapping the fixed flanking sequences.

For CUT&Tag data, paired-end reads were aligned to the hg19 genome assembly by using BWA-MEM (72). Then the peaks were called by MACS2.0 (73) with the parameter of -p 0.01. The control library was generated by treating WT 293T cells with FLAG-tag antibody. Overlap analysis was performed using bedtools (74). Enriched motifs for all types of data were discovered and visualized by HOMER program (75).

For RNA-seq data, paired-end reads were aligned to the human hg19 genome by using hisat2 (76). StringTie was used to generate gene counts which were fed into DESeq2 for expression analysis (77,78). Genes were considered differentially expressed for an adjusted *P* value < 0.05 and a log_2_ fold change > |1|. The promoter region between nucleotide positions −2000 and +500 from the transcriptional start site (TSS) were used to perform motif enrichment. Gene set enrichment analysis was performed by using clusterProfiler R package in RStudio (47).

### Quantification and statistical analysis

Datasets were assessed using GraphPad Prism 8. All numerical values are presented as means ± s.d. Statistical significance for B1H validation analyses was determined using two-sided Student's *t*-tests, while the comparison of RNA-seq read counts employed the Wilcoxon signed-rank test. For all situation, *P* value < 0.05 was considered statistically significant, *P* value < 0.01 was considered statistically extremely significant.

## Results

### Analysis of tandem repeat proteins

**Figure 1.**
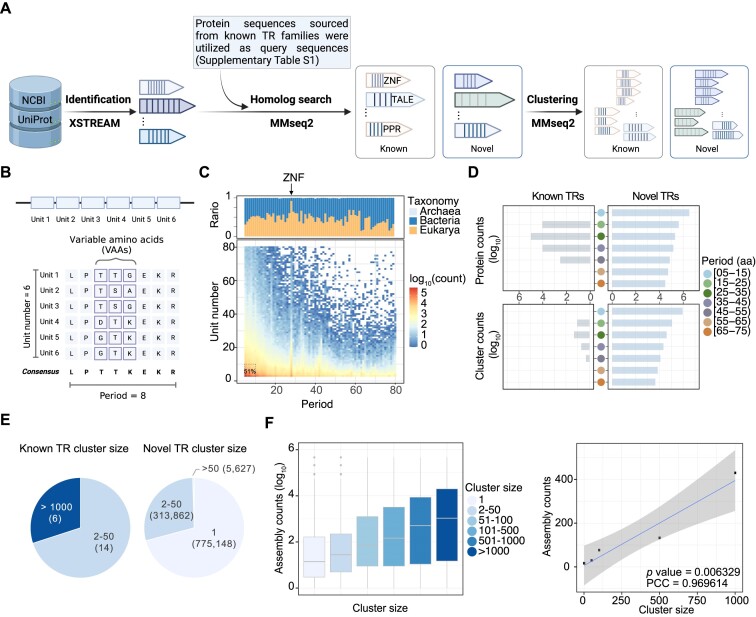
Identification and characterization of TRPs. (**A**) Computational pipeline for the identification of both known and novel TRs within the nonredundant protein database. Created with BioRender. (**B**) Schematic illustrating the TR-related terms and parameters. Created with BioRender. (**C**) Distribution of unit numbers and periods for all TRs with periods ≤ 80 and repeat units ≤ 80. Dashed box indicates the proportion of short TRs with periods ≤ 10 and repeat units ≤ 10. The upper box displays the taxonomic distribution for different periods. The arrow indicates the location of ZNFs. (**D**) Distribution of known and novel TR counts at both the protein and cluster levels. The counts of proteins and clusters were converted to log_10_ scale. TRs with different period lengths are color coded. (**E**) Cluster size distribution of known and novel TRs. Each color represents a distinct cluster size range. The absolute numbers of clusters were illustrated on the pie plot. (**F**) The left panel illustrates the distribution of cluster sizes and the counts of genus-level assemblies. Specifically, for TRs within each cluster size range, we retrieved the count of genus-level assemblies accessible at NCBI for the originating species of each protein and then depicted the corresponding distribution. The right panel represents the Pearson correlation analysis between cluster size and the median statistics for the count of genus-level assemblies.

We first developed a pipeline to identify and categorize all proteins with TR features (Figure 1A). The following terminologies are used throughout the paper: a single repeating subsequence is referred to as a ‘repeat unit’, and the length of one repeat unit is defined as the ‘period’. The count of ‘repeat unit’ is defined as the ‘unit number’. The part of the protein that includes repeats is the ‘repeat region’. The consensus sequence was derived using the majority rule. Upon aligning all repeat units, the assessment of the conservation of each column enables the derivation of variable amino acids (VAAs) (Figure 1B).

First, we searched the UniRef100 and NCBI-nr databases using the XSTREAM program with the default parameters (unit numbers ≥ 2 and period ≥ 3) and identified 43,648,764 TRs (29,79–80). TRs that exhibited the following characteristics were removed from further analysis (Supplementary Figure S1A): (i) incomplete TRs (i.e. repeat starts/ends at the protein's left/right boundaries), (ii) composite repeats defined by Perycz *et al.* (repeats built from shorter repeats, e.g. LRRLRRLRR) (81) and (iii) TRs consisting of only two repeat units. In total, 4,575,091 TRs passed this filtration, and the distribution of their periods and unit numbers showed great variability, with approximately 51% of TRs consisting a repeat period of 10 amino acids or fewer and a unit number ≤ 10 (Figure 1C). Several peaks were observed at specific period lengths, indicating a frequent distribution. Notable instances include ZNFs, which are abundantly present across all domains of life, especially in eukaryotes.

Next, we classified these TRs into known and novel categories. We identified well-studied TR families from literatures and retrieved curated proteins within these families from the UniProtKB/Swiss-Prot database (30–33) (Supplementary Table S1). The repeat region sequences of these proteins were utilized as queries to search for homologs among all identified TRs (Materials and methods). Sequences with 30% identity and 70% coverage or greater were designated as putative known TRs and subsequently confirmed by domain annotation. The selection of a 30% identity cutoff was motivated by the ability to effectively differentiate inter- and intra-repeat clusters (Supplementary Figure S1B). Impressively, among 4,575,091 TRs, only 4.4% (199,240) were categorized as known TRs, and the remaining 95.6% (4,375,851) were novel (Supplementary Figure S1C).

Next, we clustered the known and novel TRs using a 30% identity and 70% coverage cutoff (Materials and methods), and they showed distinct patterns. As indicated in Figure 1D, known TRs were grouped into a much smaller number of unique clusters, and their periods were concentrated between 15–55 aa. In comparison, the protein numbers and cluster numbers of novel TRs were much closer to each other (Figure 1D). On average, the number of proteins within each cluster was much smaller for novel TRs than for known TRs (Figure 1E). Notably, a significant proportion of the novel clusters (∼71%) consisted of only one protein member, while no orphan TRs were found among the known TR clusters (Figure 1E). The significant prevalence of orphan TRs in novel types could be attributed to limited sequencing coverage of genomic diversity or poor genome assembly quality. To identify the primary reason, we quantified two indicators across different cluster sizes: (i) the count of genus-level assemblies accessible from NCBI for the originating species of each protein and (ii) the completeness of the genome assembly to which the TRs belonged. As the cluster size increased, we observed a corresponding rise in the count of the median assembly at the genus level (Figure 1F, PCC = 0.97, *P* value < 0.01). However, the pattern of assembly completeness exhibited only a weak trend (Supplementary Figure S1D, PCC = 0.66, *P* value = 0.2285). These findings indicate that the high percentage of orphan TRs is primarily due to inadequate genome sequencing data from neighboring species, and enormously diverse novel TR families remain to be explored.

Taken together, these analyses show that only a tiny portion of TR diversity has been studied to date, and our current understanding of TRs is deeply biased toward a few well-studied families. A comprehensive exploration of TRs is needed.

### Prediction of DNA-binding TRPs

TRPs plays critical roles in binding diverse ligands, including protein, DNA, RNA and small molecules (5,82). Among these, DNA-binding TRPs not only reveal significant biological insights but also provide valuable materials for the development of gene editing tools, such like ZNF and TALE (16,83). Regarding the pivotal role of DNA-binding TRPs, our subsequent efforts were directed towards identifying TRPs possessing DNA-binding capabilities.

Initially, we employed several filtering criteria to characterize TRs with potential programmability in binding DNA sequences. Specifically, based on known nucleic acid binding families with important biological functions, we focused on repeats of 15–35 amino acids long that recur 6–40 times (15 ≤ period ≤ 35, 6 ≤ unit numbers ≤ 40) within a protein to enable proper folding with a reasonably complex binding interface. Additionally, previous studies have shown that variation between and within each repeat may empower adaptability to new environments (19,84–85). To ensure diversity within clusters, we excluded clusters if the repeat regions of all members were identical. To assess within-repeat diversity, the normalized Shannon's entropy score (NSS) was introduced by calculating the amino acid diversity of the consensus sequence (Materials and methods). In total, 125,624 TRs with a defined period and unit number, as well as an NSS exceeding 0.6, passed the filtration procedure and were named ‘TRs of interest’ (Supplementary Figure S1C and Supplementary Table S2). We developed a hierarchical naming scheme for the ‘TRs of interest’ proteins that incorporates several levels of classification: period, cluster size and unit number (Supplementary Figure S1E).

For predicting DBPs, multiple computational techniques have been developed by incorporating diverse information sources such as sequences, structural features, and physicochemical properties (86–92). We conducted a comprehensive analysis of all available DBP prediction models developed since 2010 and found that 78% of the models were based on traditional machine learning (TML), while 22% were based on deep learning (DL) (Supplementary Table S3). Furthermore, we found that TML-based models tended to have smaller training datasets compared to DL-based models (Supplementary Table S3). Notably, approximately 67% of TML models utilized the PDB1075 dataset (91), whose limitations have recently been reported (93). Therefore, our endeavors concentrated on two aspects: (i) generating high-quality datasets and (ii) developing a robust model.

**Figure 2.**
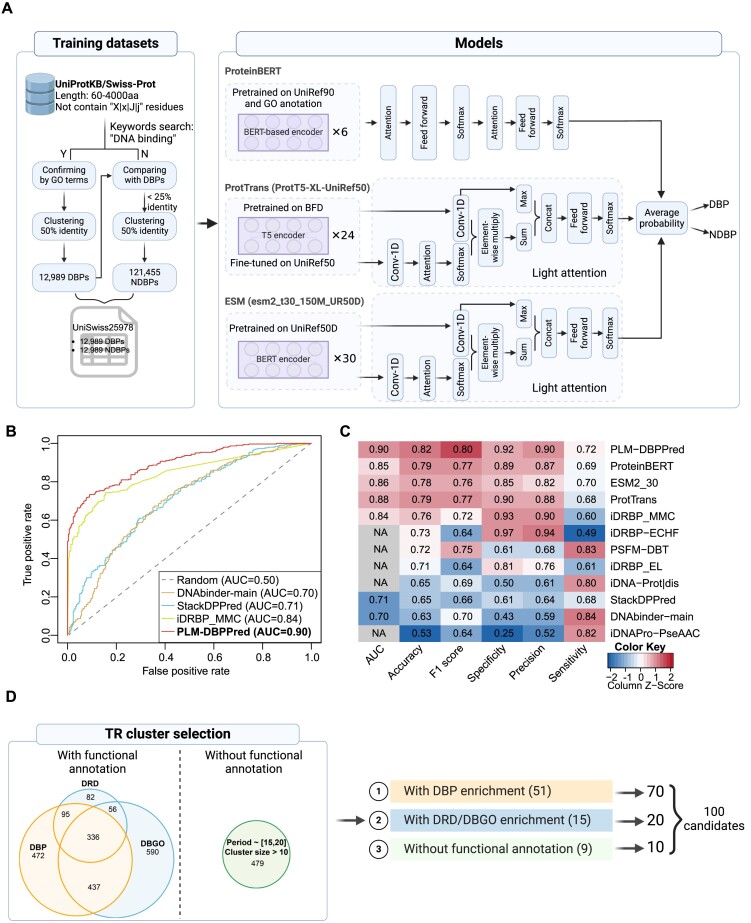
DBP prediction model and strategy of candidate prioritization. (**A**) The generation of training datasets and PLM-DBPPred architecture integrates three PLM models (ProteinBERT, ProtTrans and ESM) to predict DBPs. Created with BioRender. (**B**) Comparing PLM-DBPPred with other DBP classifiers via ROC-AUC analysis utilizing the PDB600 test dataset. AUC values are indicated for each classifier. (**C**) Evaluation results of various DBP prediction tools. Each column was individually normalized, and the performance is depicted using a color key. (**D**) Strategies to select TR clusters and candidates. The Venn plot illustrates the enriched cluster list generated by different functional annotation methods, wherein DBP, DRD and DBGO represent DNA binding proteins predicted by PLM-DBPPred, DNA-related domains and DNA binding-related GO annotations, respectively. Created with BioRender.

Firstly, we assembled a high-quality training dataset from the UniProtKB/Swiss-Prot database, encompassing 12,989 DBPs and an equal number of NDBPs, collectively referred to as the UniSwiss25978 dataset (version 2022.10, Figure 2A). Additionally, an independent test dataset, named PDB600, was generated, consisting of 300 DBPs and 300 NDBPs sourced from the PDB database. Subsequently, we investigated three prominent PLMs: ProteinBERT (24), ProtTrans (25) and ESM (26), each distinguished by unique architectures and pre-training on diverse databases. We integrated these PLMs with various classification layers. For the ProteinBERT-based model, we evaluated two architectures: one incorporating two attention layers, and another integrating only fully connected layers. For the ProtTrans-based and ESM-based models, we combined each with three distinct classifiers: MLP, light attention (LA) (44), and biLSTM_TextCNN (45). Finally, probabilities were obtained for each model through a feed-forward network (Materials and methods). We fine-tuned these models using the UniSwiss25978 dataset and subsequently evaluated their performance using the PDB600 dataset, as presented in Supplementary Table S4.

Based on the test results, the optimal combination for each language model was selected, namely ProteinBERT with two attention layers, ProtT5 with LA classifier, and ESM2_30 with LA classifier (Supplementary Table S4). As depicted in Supplementary Figure S2A, ROC–AUC analysis illustrated robust predictive capabilities of all three models in identifying DBPs, and the ProtTrans-based model generated the best outcomes, with an impressive AUC of 0.88. To further improve model performance, we adopted an ensemble learning strategy to integrate all three models. Intriguingly, combining these models enhanced classification performance, resulting in an AUC score of 0.90 (Figure 2B). This newly developed transformer-based model combining three PLMs was designated PLM-DBPPred (protein language model-enhanced DNA binding protein prediction) (Figure 2A). Next, we compared it with existing state-of-the-art methods (86–92,94–95). PLM-DBPPred showed the best performance, demonstrating superior performance in AUC, accuracy and F1 score, with values of 0.90, 0.82 and 0.80, respectively (Figure 2C).

Using PLM-DBPPred, we predicted DBPs for the full-length proteins of the ‘TRs of interest’ dataset, resulting in a candidate list that consists of 8,865 TRPs, distributed across 1,640 clusters, with potential DNA-binding capability.

### Selecting TRP candidates for experimental screening

To further prioritize candidates with a higher likelihood of being DBPs, we conducted a survey of the well-studied DBPs to gain deeper insights into the biological processes in which they participate and their domain characteristics. We first performed Gene Ontology (GO) analysis using a dataset containing 1,000 DBPs and 1,000 NDBPs in the PDB database. As expected, DBPs were highly enriched in DNA-related metabolism and functions compared to NDBPs (Supplementary Figure S2B). According to the enriched GO terms, we traced them to higher-level hierarchy, including GO:0090304 (nucleic acid metabolic process), GO:0003700 (DNA-binding transcription factor activity), GO:0003676 (nucleic acid binding), GO:0140640 (catalytic activity, acting on a nucleic acid), and GO:0005634 (nucleus) (Supplementary Figure S2B). TRPs with annotations to these terms and their subterms were categorized into the DNA-binding related GO (DBGO) list.

Considering that DBPs are often composed of DNA binding domains (DBDs) and specific functional domains, the annotation of partner domains will facilitate the identification of DBDs (96,97). Therefore, we conducted an analysis of the partner domains associated with all previously identified DBDs and found that they were enriched in nucleic acid metabolic processes and occurred in various forms, such as hydrolases, nucleases, and methyltransferases (Supplementary Figure S2C). We then generated a DNA-related domain (DRD) collection (Supplementary Table S5), and TRPs annotated with these domains were categorized into the DRD list.

Given that proteins grouped in clusters often share similar functional annotations and features, we established several selection strategies at the cluster level. For strategies based on functional annotation, we calculated an enrichment score for each functional annotation (Materials and methods). A cluster with a specific function was designated when the corresponding log_2_ odds ratio (LR) score exceeded 0 (52). Accordingly, an overlap cluster between predicted DBP, DBGO and DRD was generated (Figure 2D). We first selected 51 clusters with DBP function enrichment. We next selected 15 clusters with DRD/DBGO functional enrichment. In addition, considering that all of these functional annotations relied on existing knowledge, we further selected nine clusters without any functional annotation and based solely on basic protein features such as small size. Due to the variations in cluster sizes and the difficulty of synthesizing genes containing tandem repeats, a variable number (1–3) of proteins within each cluster were chosen for gene synthesis (Supplementary Table S6). Overall, genes encoding 100 members distributed in 75 clusters were successfully synthesized. It is worth noting that in some cases when the protein contains DRDs, only gene fragment encoding the repeat region was synthesized.

### Experimental screening and validation

**Figure 3.**
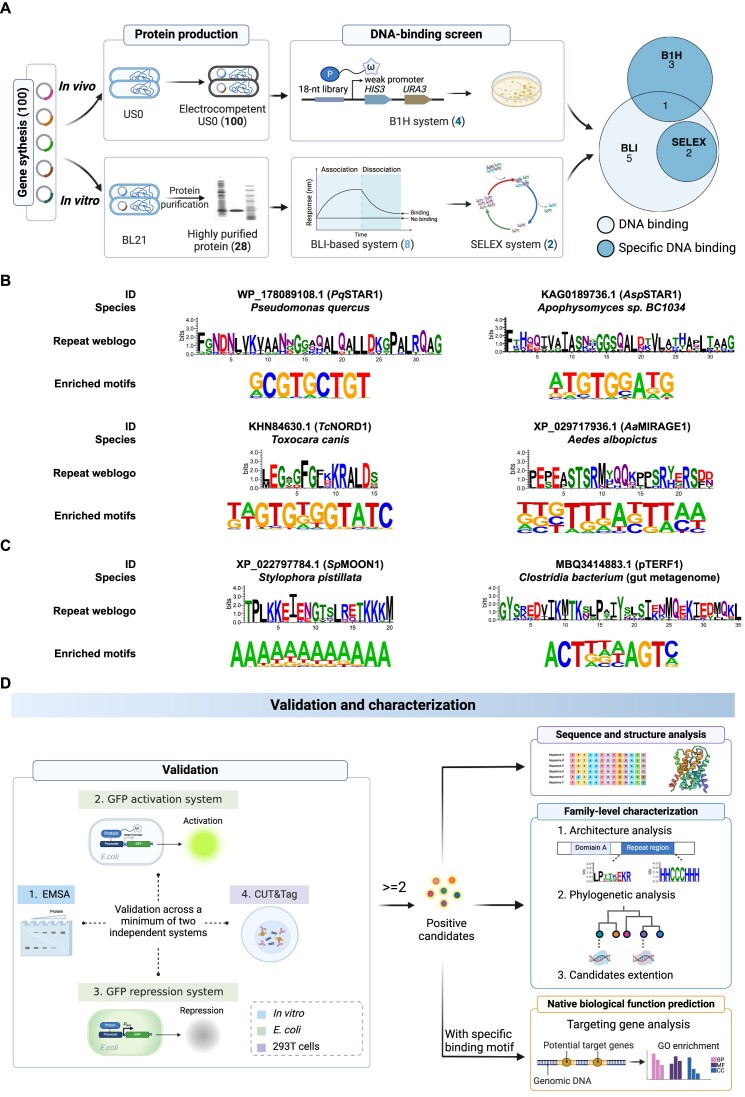
Experimental screening and validation design. (**A**) Experimental screening pipeline. For the *in vivo* B1H screen, all 100 candidate genes were cloned into B1H protein expression vectors and subsequently screened. For the *in vitro* screen, all 100 candidate genes were initially assessed for their expression levels in *E. coli*. Subsequently, proteins with high expression were purified. The purified proteins were then subjected to a BLI-based screening to test their DNA binding activity. Candidates showing DNA-binding activities were further subjected to SELEX, the enriched libraries were sequenced, and the enriched binding motifs were obtained. The numbers in each step indicate the corresponding candidates identified and selected during the screening process. The Venn plot illustrates the positive candidates identified by different screening methods. The light blue and dark blue colors represent positive candidates with DNA binding activity and specific DNA binding activity, respectively. Created with BioRender. (**B**) Positive candidates identified by the B1H platform. For each candidate, we provide information including the protein accession (a customized name was given to each, shown in parenthesis), resource species, repeat weblogo and enriched binding motif. (**C**) Positive candidates identified by the SELEX platform. (**D**) Experimental design for the validation and characterization of positive candidates. We established four independent validation methods to verify protein binding activity toward the enriched motifs. These methods included one *in vitro* platform (EMSA), two GFP-based platforms in *E. coli*, and the CUT&Tag platform in 293T cells (Materials and methods). Proteins that were validated in two or more independent assays were deemed true positives. The analysis of positive candidates was then extended to three further analyses: sequence and structure feature analysis, family-level characterization and native biological function prediction. Created with BioRender.

Considering the unique characteristics of different DBPs and their variable compatibility with specific functional assays, we employed both *in vivo* and *in vitro* screening strategies for identifying novel DNA-binding TRPs (Figure 3A).

For the *in vivo* platform, the well-established B1H system was employed, which converts DNA binding events into the survival of bacteria (98). We cloned all 100 candidate genes into the B1H vector and performed screening as previously described (98). Among all 100 candidates, we successfully identified specific binding motifs for four different proteins (Figure 3A, B).

For the *in vitro* platform, we first assessed the expression level of each protein in *E. coli* and further purified the proteins with high expression. Out of the 100 candidates, we produced 28 proteins with high purity (Figure 3A and Supplementary Figure S3A). Subsequently, we developed a BLI-based screening method, validated the method by comparing the responses of well-known DBPs and NDBPs, and determined the appropriate experimental conditions (Materials and methods and Supplementary Figure S3B). Accordingly, we conducted BLI-based screen for 28 purified candidates, and identified eight proteins that displayed DNA binding activity under a 0.1 nm binding response (nm shift in BLI curve) cutoff (Supplementary Figure S3C). Next, we performed SELEX analysis (99) of these eight candidates to determine their binding specificity and successfully identified enriched DNA sequence motifs for two candidates (Figure 3A, C).

In total, we identified 11 proteins with DNA-binding activity, six of which displayed sequence specificity of DNA binding under the specific screening conditions (Figure 3A). To further validate the specific binding activity of these six positive hits obtained from the screening, we implemented four different validation assays (Figure 3D). To validate protein-DNA interactions *in vitro*, EMSAs were used (100). Two other methods were modified from published studies (67,98), in which protein binding to a specific DNA sequence induces or represses GFP signal in *E. coli* (Materials and methods). Additionally, CUT&Tag technology was used to identify specific DNA binding in mammalian cells (101). All six positives were validated in two or more independent assays and were therefore deemed true positives, and a customized name was given to each based on their features (Figure 3B, C), which will be described in later sections.

For each protein, we analyzed the sequence features, predicted the tertiary structure by Alphafold2 (53,102) and identified homologs. We then characterized the common features for each family, including the verification of repeat boundaries and overall protein architecture, visualization of repeat units and secondary structure patterns, and construction of a phylogenetic tree. In certain cases, additional homologs were synthesized to further elucidate the binding properties of the family (Figure 3D). In the following sections, we introduce each in detail.

### DNA-binding TRPs identified by B1H screening

#### Characterization of STAR proteins

**Figure 4.**
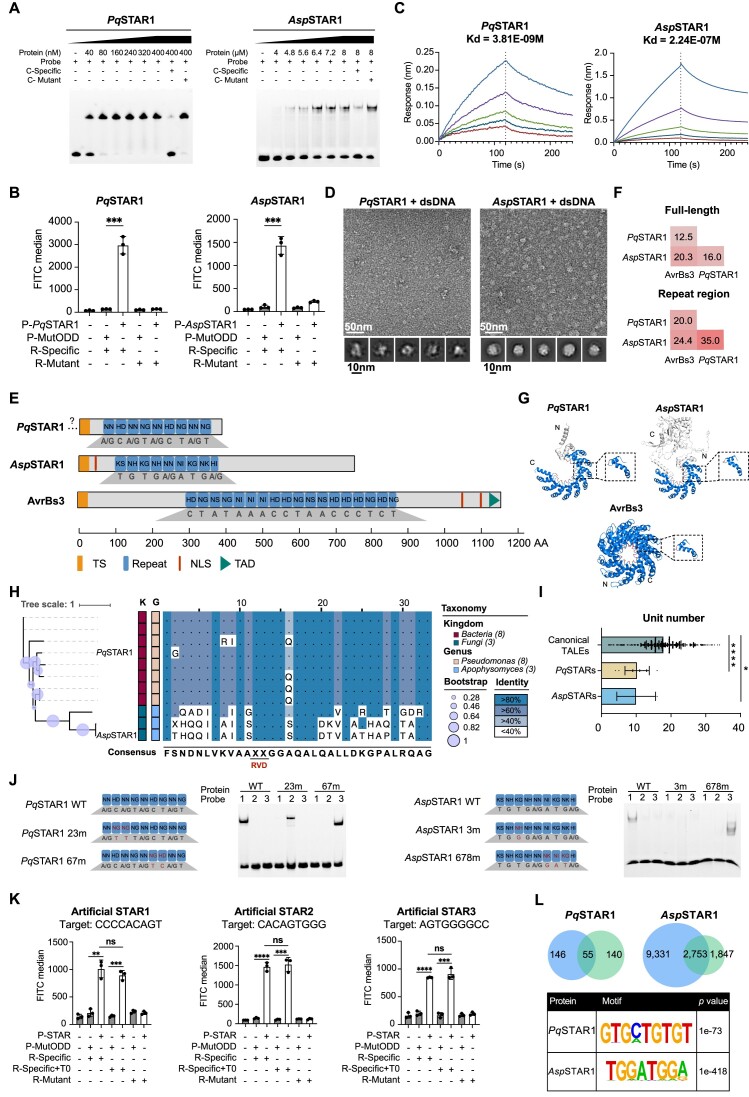
Characterization of the STAR family. (**A**) EMSA validation results for *Pq*STAR1 and *Asp*STAR1. The protein concentration was varied, while the probe content was kept constant at 40 nM. The abbreviation ‘C’ denotes the competitor probe, which was used at a concentration 100-fold higher than that of the specific probe. (**B**) GFP activation results for *Pq*STAR1 and *Asp*STAR1. MutODD is a non-DNA binding protein that served as a negative control. ‘P’ and ‘R’ represent protein and reporter plasmids, respectively. Two-sided Student's *t*-test, *n* = 3 biologically independent samples. Data are shown as mean ± s.d. ***P value < 0.001. Data were graphed in GraphPad Prism 8. (**C**) BLI binding results for *Pq*STAR1 and *Asp*STAR1. The *Pq*STAR1 protein concentrations were 0.25, 0.5, 1, 2 and 4 nM, and the *Asp*STAR1 protein concentrations were 3.125, 6.25, 12.5, 25 and 50 nM. Data were graphed in GraphPad Prism 8. (**D**) EM micrographs of negatively stained *Pq*STAR1-DNA complexes, *Asp*STAR1-DNA complex and their representative 2D classification averages. Upper panel scale bar is 50 nm, bottom panel scale bar is 10 nm. (**E**) Protein architecture of *Pq*STAR1, *Asp*STAR1 and AvrBs3. TS, type III secretion signal. NLS, nuclear localization signal. TAD, transcriptional activation domain. The gene encoding the predicted *Pq*STAR1 lacks a start codon; we therefore placed a ‘?’ at the N-terminus of STAR1. (**F**) Sequence comparison of *Pq*STAR1, *Asp*STAR1 and AvrBs3. (**G**) Protein tertiary structure of *Pq*STAR1, *Asp*STAR1 and AvrBs3. Structures of *Pq*STAR1 and *Asp*STAR1 were predicted by AlphaFold2. The structure of AvrBs3 was derived from the PDB database (2YPF). N and C represent N- and C-terminal ends. The repeat region and RVDs are colored blue and red, respectively. The expanded dashed box illustrates the structure of a single repeat unit. (**H**) Multiple sequence alignment and phylogenetic tree of *Pq*STAR homologs and *Asp*STAR homologs. The phylogenetic tree was constructed using full-length protein sequences. In each aligned column, the degree of conservation is represented by the undertone of the amino acid. The bootstrap confidence scores are represented by circle sizes. Squares with different colors in the middle represent different taxa. The color and identity coordinates are shown on the right. (**I**) Distribution of unit numbers of canonical TALEs, *Pq*STARs and *Asp*STARs. canonical TALEs represent TALEs from *Xanthomonas*, *Pq*STARs represent STARs from *Pseudomonas*, and *Asp*STARs represent STARs from *Apophysomyces*. Statistical analysis was by one-way ANOVA analysis in GraphPad Prism. *P value < 0.05; ****P value < 0.0001. (**J**) Reprogramming DNA binding specificity of *Pq*STAR1 and *Asp*STAR1. For *Pq*STAR1, WT, 23m and 67m represent the wild-type protein, a variant with modified RVD2 and RVD3 and a variant with modified RVD6 and RVD7, respectively. For *Asp*STAR1, WT, 3m and 678m represent the wild-type protein, a variant with modified RVD3, and a variant with modified RVD6, RVD7 and RVD8, respectively. RVD alterations are highlighted in red. The nucleic acid bases under the corresponding RVDs are derived following the binding code of canonical TALEs. In the EMSA assay, probe 1 corresponds to the target sequence of the wild-type protein, while probes 2 and 3 represent the target sequences of the two variants. (**K**) GFP activation results for artificial STARs. MutODD is a non-DNA binding protein serving as a negative control. ‘P’ and ‘R’ represent protein and reporter plasmids, respectively. Two-sided Student's *t*-test, n = 3 biologically independent samples. Data are shown as mean ± s.d. **P value < 0.01; ***P value < 0.001; ****P value < 0.0001. Data were graphed in GraphPad Prism 8. (**L**) CUT&Tag results of *Pq*STAR1 and *Asp*STAR1 in 293T cells. Two biologically independent samples were employed.

Through B1H screening, we identified DNA motif enrichment for two proteins within the same cluster, namely, *Pq*STAR1 and *Asp*STAR1 (Figure 3B). Subsequently, we confirmed their specific binding to the identified DNA sequences through EMSA and GFP activation assays (Figure 4A, B). The binding affinities of *Pq*STAR1 and *Asp*STAR1 were further quantified using BLI assay and found to be approximately 3.8 and 224 nM, respectively (Figure 4C). Notably, the negative stain electron microscopy (EM) analysis revealed a more uniform protein structure in the presence of DNA, as compared to the condition with protein alone (Figure 4D and Supplementary Figure S4A). The 2D classification results revealed an average particle size of approximately 120 Å and 240 Å for *Pq*STAR1-DNA complex and *Asp*STAR1-DNA complex, respectively (bottom panel of Figure 4D).

Comparative analysis of these sequences revealed some degree of conservation at specific amino acid positions (Figure 3B). Intriguingly, we observed high variability at positions 12 and 13, similar to the repeat variable di-residues (RVDs) of TALE-like repeats (Figure 3B). Therefore, we conducted a comparative analysis between the two newly identified proteins and canonical TALEs (AvrBs3 was chosen as a reference for comparison). The overall architectures of *Pq*STAR1, *Asp*STAR1 and AvrBs3 shared certain features, such as the type III secretion signal (T3SS) and a central DNA-binding repeat domain (Figure 4E). However, differences were evident: the lengths of the whole proteins and the nonrepetitive regions of *Pq*STAR1 and *Asp*STAR1 were shorter (note: the gene encoding the predicted *Pq*STAR1 lacks a start codon), there were fewer repeat units, and both lacked a C-terminal transcriptional activation domain. Pairwise comparisons across full-length proteins and repeat regions revealed low sequence identity with canonical TALE (Figure 4F), which explained why these two proteins were not initially identified as known TRs. Multiple sequence alignment showed that only nine positions out of 34 were highly conserved across all three proteins (Supplementary Figure S4B), while the RVDs of *Pq*STAR1 and *Asp*STAR1 were largely the same as those of canonical TALEs, and the DNA motif recognized by each protein correlated well with the order of RVDs (Figure 4E), indicating that they shared the TALE code of nucleotide sequence recognition.

Next, we subjected these two proteins to protein disorder and structure prediction using DISOPRED3 and AlphaFold2 (53,102,103). The repeat regions exhibit well-ordered structures, with an average pLDDT (predicted local distance difference test) score exceeding 80, indicating a confident 3D conformation (Supplementary Figure S4C). The overall structures exhibit a helix-loop-helix architecture, with the RVD positions within the loop region, resembling the canonical TALE structure represented by AvrBs3 (Figure 4G). Notably, quantification using BLI indicated that *Pq*STAR1 exhibited a high DNA-binding affinity with only 9 repeats (*K*_d_ = 3.8 nM, Figure 4C), surpassing that of the canonical TALE with 10 repeats (*K*_d_ = 400 nM) by 100-fold (104). These data collectively suggest that despite some similarity in the overall structure, these two proteins distantly related to TALEs show a higher or comparable DNA-binding affinity, even with a lower unit number. Hence, we designated these two proteins as **S**hort **TA**LE-like **R**epeat proteins (STAR), named *Pseudomonas quercus* STAR homolog 1 (*Pq*STAR1) and *Apophysomyces sp*. STAR homolog 1 (*Asp*STAR1), respectively, based on the species of origin and the order of characterization.

Subsequently, we identified all homologous proteins within the originating genus of *Pq*STAR1 and *Asp*STAR1. Overall, we identified seven homologs with clear repeat boundaries for *Pq*STAR1 and two for *Asp*STAR1 (Supplementary Table S7). We constructed an evolutionary tree using the full-length sequences of these proteins (Figure 4H). The consensus sequence alignments revealed a high degree of amino acid conservation within groups, particularly among *Pq*STARs (Figure 4H). Intriguingly, compared to the canonical TALEs, these two types of STAR proteins overall had smaller number of repeats (Figure 4I), suggesting different DNA binding properties.

The identification of clear binding sequences motivated us to search for potential target genes of *Pq*STAR1 and *Asp*STAR1. The T3SS prediction suggested that *Pq*STAR1 and *Asp*STAR1 can be secreted by type III secretion system, implying a possible role in the host genome. The originating species of *Pq*STAR1, *Pseudomonas quercus*, was extracted from *Quercus mongolica* leaf spots, suggesting *Quercus mongolica* as a potential host (105). We performed GO enrichment analysis of potential target genes in original genome (*Pseudomonas quercus*) and potential host genome (*Quercus mongolica*) (Supplementary Figure S4D). Interestingly, enrichment of GO terms is exclusively observed within the gene set of the host genome, predominantly participating in stress response processes, which suggests *Pq*STAR1’s role as an effector in regulating host stress-related genes. Following the recognition patterns of RVDs, we conducted a similar analysis of the potential target genes for other three *Pq*STARs (Supplementary Table S7 and Supplementary Figure S4D). The results were consistent with those observed for *Pq*STAR1, strongly suggesting that *Pq*STARs participate in the infection process of *Pseudomonas quercus* on *Quercus mongolica* by regulating host stress-related genes. Another STAR protein, *Asp*STAR1, was derived from *Apophysomyces sp. BC1034*, representing the first TALE-like protein identified in eukaryotic species. However, the analysis of potential *Asp*STAR1 target genes was unfeasible due to the lack information of known hosts.

To test the DNA-binding specificity of *Pq*STAR1 and *Asp*STAR1, we designed two variants of each by modifying the RVDs of one to three repeat units (Figure 4J). Both EMSA and GFP activation assays indicated that *Pq*STAR1 RVD variants showed high specificity to new target sequences with only two RVDs modified (Figure 4J and Supplementary Figure S4E). The *Asp*STAR1 RVD variant showed similar specific binding activity, albeit with lower activity. Due to the high specificity and strong affinity of *Pq*STAR1 in binding DNA, we aimed to assess its potential as a programmable DNA binding tool. To this end, we first constructed a collection of plasmids encoding diverse repeat modules derived from the *Pq*STAR1 backbone and utilized the Golden Gate method to assemble customized repeat arrays (70). Subsequently, we assembled three artificial STAR proteins targeting different sequences, each comprising nine repeat units. In the GFP activation assay, all artificial STARs activated GFP expression, and exhibited binding activity independent of the 5′ thymine (T0) at the binding site (Figure 4K), which is preferred by TALEs (106). Collectively, these findings indicate that *Pq*STAR1 can be readily designed to target specific DNA sequences without T0 dependency.

Next, we expressed *Pq*STAR1 and *Asp*STAR1 with a 3X FLAG tag in 293T cells and conducted a CUT&Tag assay. Western blotting indicated that both *Pq*STAR1 and *Asp*STAR1 were expressed efficiently (Supplementary Figure S4F). Furthermore, the motifs enriched in the CUT&Tag assay were similar to both the B1H screen results and the predicted motifs (Figure 4L). These data collectively indicate that *Pq*STAR1 and *Asp*STAR1 bind to specific DNA sequences in the human genome, supporting their potential application in human cells.

#### Gene activation by STAR-based transcriptional regulators

Artificial transcription factors (ATFs) are DNA binding regulators designed to control the expression of a specific gene or a set of genes in a predetermined manner (66,107–109). While ATFs based on TALE and CRISPR have been developed, they usually regulate one target gene, due to the relatively long target sequence required for binding (110–112).

To compare the capability of STAR and TALE in binding short sequence motifs, we first constructed artificial STARs and TALEs targeting the binding motifs of two well-known TFs, namely NF-κB and SMAD4 (113). Both the gene activation assay and the EMSA assay indicated that the canonical TALE-based ATFs containing only nine repeats lack binding activity (Supplementary Figure S5). In contrast, STAR-based ATFs bind to 9 bp target sequences effectively (Supplementary Figure S5), demonstrating the unique advantages of STARs in binding short DNA motifs.

**Figure 5.**
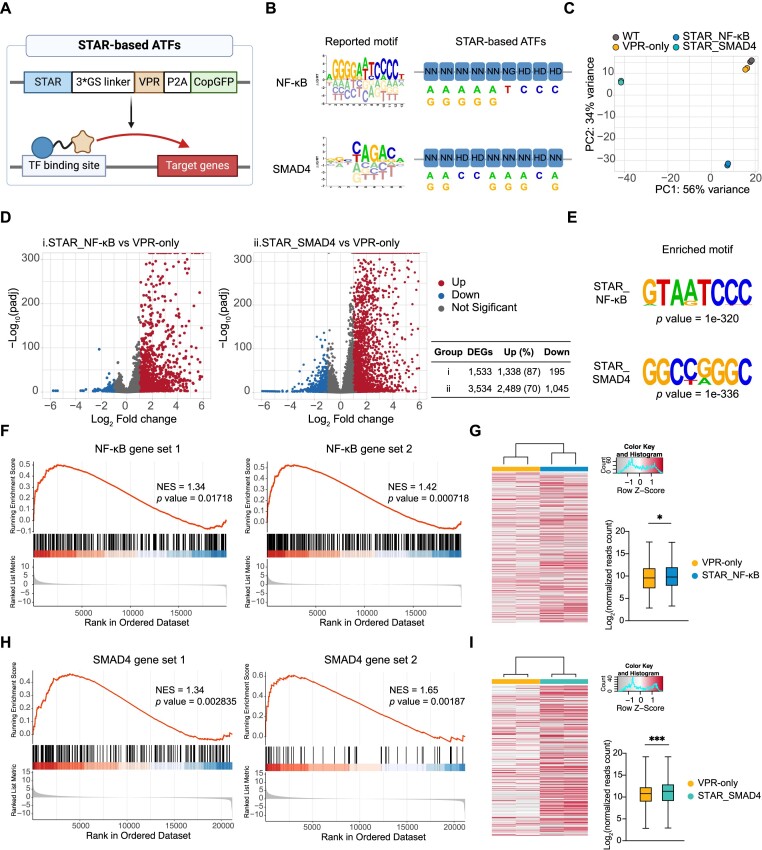
Characterization of STAR-based transcriptional regulator. (**A**) Illustration of STAR-based transcriptional regulator. Created with BioRender. (**B**) Reported TF binding motif and RVD design of STARs. (**C**) Principal component analysis (PCA) for RNA-seq samples. PCA was performed using rlog-transformed count data from DESeq2. (**D**) Significantly up-regulated (red) and down-regulated (blue) transcripts after STAR-based ATFs transduction (*P* value < 0.05 and a log_2_foldchange > |1|). (**E**) Motifs enriched in the up-regulated gene promoter regions. (**F**) Gene set enrichment analysis (GSEA) plots of 2 previously characterized NF-κB regulated gene sets. Gene set 1 and 2 derived from Cormier *et al.* (116) and the TFlink database (115), respectively. NES, normalized enrichment score. (**G**) Heatmap of normalized reads count for genes listed in (F) (combination of gene set 1 and 2). Statistical significance analysis was employed by the Wilcoxon signed-rank test in GraphPad Prism. *P value < 0.05, **P value < 0.01. (**H**) Gene set enrichment analysis (GSEA) plots of two previously characterized SMAD4 regulated gene sets. Gene sets 1 and 2 resourced from the MSigDB database (SMAD4_Q6) (114) and the TFlink database (115), respectively. (**I**) Heatmap of normalized reads count for genes listed in (H) (combination of gene sets 1 and 2). Statistical significance analysis was employed by the Wilcoxon signed-rank test in GraphPad Prism. **P value < 0.01; ***P value < 0.001.

Next, we fused the aforementioned STARs with VPR activation domain at the C-terminus, expressed them in human 293T cells (Figure 5A, B), and performed RNA-seq analysis for four groups of samples, including a wild-type (WT) group without plasmid transduction, the VPR-only group (VPR-only), STAR targets NF-κB binding sites (STAR_NF-κB) and STAR targets SMAD4 binding sites (STAR_SMAD4). A robust reproducibility was observed across biological replicates, wherein the VPR-only group closely resembling the WT group (Figure 5C). In contrast, the groups treated with STAR-based ATFs exhibit a discernible deviation from the control group, highlighting distinctive transcriptional alterations induced by STAR-based ATFs (Figure 5C). Compared to the VPR-only group, transfection with STAR_NF-κB and STAR_SMAD4 resulted in the up-regulation of 1,338 and 2,489 differentially expressed genes (DEGs, *P*_adj_ < 0.05 and fold change > 2), accounting for the majority of DEGs (> 70%) (Figure 5D).

Next, we performed motif enrichment and gene set enrichment analysis (GSEA) to determine whether these up-regulated DEGs are truly targets of the designed STAR-based ATFs. Notably, the promoter region of these up-regulated DEGs exhibited enrichment for motifs closely resembling the binding motifs of NF-κB and SMAD4 (Figure 5E). Moreover, previously published gene sets of NF-κB/SMAD4-targeted genes were significantly enriched in the up-regulated genes of STAR-based ATF groups (114–116) (Figure 5F, H). The expression levels of the reported target genes were significantly higher than those in the VPR-only groups (Figure 5G, I). Collectively, these data indicated that STAR-based ATFs effectively enhanced the expression of a large number of endogenous genes by targeting a specific regulatory motif shared by these genes, establishing a proof of concept for STAR as a platform for constructing ATFs that regulate the transcription network.

#### Other candidates identified by B1H screening

In addition to *Pq*STAR1 and *Asp*STAR1, two other candidates (KHN84630.1 and XP_029717936.1) exhibited enrichment of specific motifs in the B1H screening. Since we were not able to purify these two proteins for *in vitro* analysis, their binding activity was subsequently verified through two independent *in vivo* functional validation assays (Supplementary Figure S6A, S7A).

Notably, KHN84630.1, from *Toxocara canis*, has a small size, with a period length of only 15 aa (Figure 3B). Accordingly, we named it **N**emat**O**de **R**epeat **D**NA binding protein (NORD), specifically denoted as *Toxocara canis* NORD homolog 1 (*Tc*NORD1). For tertiary structure prediction, the average pLDDT score was lower than 50, with observed high disorder scores in certain regions (Supplementary Figure S6B, C). Next, we expanded the scale of analysis to the family level and found 55 *Tc*NORD1 homologs in species distributed primarily in two orders: *Rhabditida* and *Strongylida* (Supplementary Figure S6D, E and Supplementary Table S8). Moreover, sequences in the *Rhabditida* genus are more diverse than those in *Strongylida*. Notably, one of the homologs, originating from *Caenorhabditis elegans*, was encoded by neuropeptide-like protein 14 (*nlp-14*). Previous studies have reported *nlp-14*′s role in modulating cholinergic signaling during male mating, mediating decision-making during nociceptive behaviors and regulating sleep (117–119), whereas no DNA binding activity has been suggested. The discovery of DNA binding activity of *Tc*NORD1 may offer a fresh perspective on the functionality of neuropeptide-like proteins.

Another candidate, XP_029717936.1, from *Aedes albopictus*, enriched a more degenerated binding motif (Figure 3B). Accordingly, we named it **M**osqu**I**to **R**epeat DN**A** bindin**G** prot**E**in (MIRAGE), specifically denoted as *Aedes albopictus* MIRAGE homolog 1 (*Aa*MIRAGE1). The tertiary structure prediction score was low, and the repeat region was highly disordered as well (Supplementary Figure S7B, C). Domain analysis indicated that *Aa*MIRAGE1 contains two RNA-binding motifs (RRMs) that are known to interact with RNA and DNA as well as proteins (120) (Supplementary Figure S7D). Since we only synthesized the sequence encoding the repeat region of this protein for screening, the detected DNA binding motifs were not influenced by the RRMs. Interestingly, potential genes targeted by *Aa*MIRAGE1 may play a role in trehalose metabolism and calcium ion transport processes, suggesting a potential functional connection of *Aa*MIRAGE1 with energy metabolism and signal transduction (Supplementary Figure S7E). We next retrieved homologs from the cluster to which *Aa*MIRAGE1 belongs and identified three homologs, with unit numbers ranging from 7 to 21, and sequence highly conserved (Supplementary Figure S7F, G and Supplementary Table S9). In addition to the conserved TRs, two RRMs and a NonA/paraspeckle domain (NOPS) were also conserved (Supplementary Figure S7D). Previous studies have reported a comparable architectural framework in the Drosophila behavior/human splicing (DBHS) family, which acts as a versatile molecular scaffold through interacting with DNA, RNA and proteins (121). Interestingly, MIRAGE proteins and DBHS proteins only shared homologies with the RRM and NOPS domain, while the N-terminal sequence presented low identity (Supplementary Figure S7H). Nevertheless, one member of the human DBHS protein family, the splicing factor proline/glutamine-rich (SFPQ, a.k.a. PSF) protein, has been documented to bind to DNA (122,123). The specific DNA binding activity of *Aa*MIRAGE1 may expand the diversity of substrates for DBHS-like proteins.

Notably, the predicted tertiary structures of *Tc*NORD1 and *Aa*MIRAGE1 both displayed low confidence (Supplementary Figure S6C, S7C), likely due to the high proportion of disordered regions within the repeat sequences, consistent with the previously reported limitations of AlphaFold2 in predicting intrinsically disordered proteins (124).

### DNA-binding TRPs identified by SELEX

#### Characterization of MOON proteins

**Figure 6.**
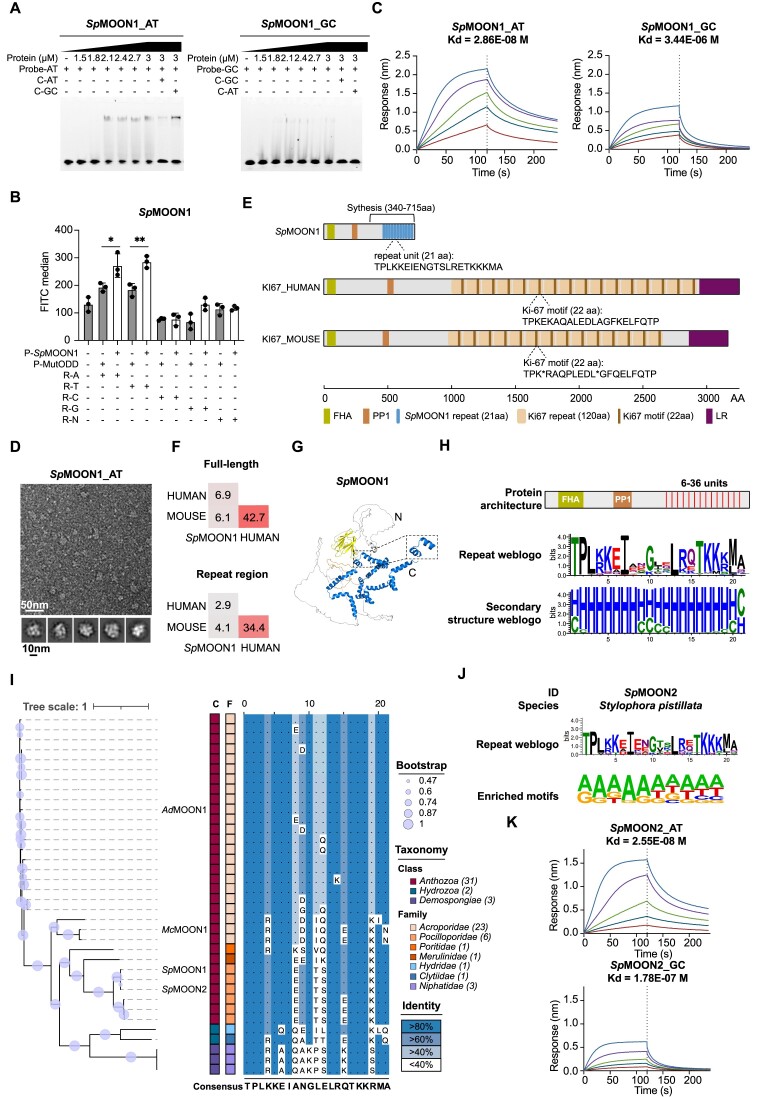
Characterization of the MOON family. (**A**) EMSA results for *Sp*MOON1. The GC-rich sequence served as a control. The protein concentration was varied, while the probe content was kept constant at 40 nM. (**B**) GFP activation validation for *Sp*MOON1. MutODD is a non-DNA binding protein that served as a negative control. ‘P’ and ‘R’ represent protein and reporter plasmids, respectively. Two-sided Student's *t*-test, *n* = 3 biologically independent samples. Data are shown as mean ± s.d. *P value < 0.05; **P value < 0.01. Data were graphed in GraphPad Prism 8. (**C**) BLI results for *Sp*MOON1. Protein concentrations were set as follows: 9.8, 14.8, 22.2, 33.3, 50 nM. Data were graphed in GraphPad Prism 8. (**D**) An EM micrograph of negatively stained *Sp*MOON1-DNA complex and its representative 2D classification averages. Upper panel scale bar is 50 nm, bottom panel scale bar is 10 nm. (**E**) Protein architecture of *Sp*MOON1, KI67_HUMAN and KI67_MOUSE protein. The architecture of KI67_HUMAN and KI67_MOUSE was derived from the literature (150). (**F**) Sequence comparison of *Sp*MOON1, KI67_HUMAN and KI67_MOUSE. (**G**) Predicted protein tertiary structure of *Sp*MOON1. The FHA, PP1_bind domain and repeat region are colored yellow, orange and blue, respectively. The expanded dashed box illustrates the structure of a single repeat unit. (**H**) Domain architecture, repeat and secondary structure weblogos of the MOON family. The functional domains, FHA and PP1_bind, are illustrated. The C-terminal region, indicated by the red rectangle, contains repeat units. The unit number within the MOON family ranges from 6 to 36. (**I**) Multiple sequence alignment and phylogenetic tree of the proteins in the MOON family. The phylogenetic tree was constructed using full-length protein sequences. The names of experimentally tested homologs of *Sp*MOON1 are labeled. In each aligned column, the degree of conservation is represented by the undertone of the amino acid. The bootstrap confidence scores are represented by circle sizes. Squares with different colors in the middle represent different taxa. The color and identity coordinates are shown on the right. (**J**) Species, repeat logos and enriched motifs of *Sp*MOON2. (**K**) Assessment of *Sp*MOON2 binding to AT/GC-rich probes using BLI assay. Protein concentrations were set as follows: 9.8, 14.8, 22.2, 33.3, 50 nM. Data were graphed in GraphPad Prism 8.

The *in vitro* screening identified two proteins that bind to specific DNA sequences. One of them (XP_022797784.1), identified from *Stylophora pistillata*, showed a binding preference toward AT-rich sequences (Figure 3C). Accordingly, we named it **M**arine **O**rganism-**O**riginated D**N**A binding protein (MOON), with the specific name *Stylophora pistillata* MOON homolog 1 (*Sp*MOON1). We first confirmed its binding activity of the enriched motif by EMSA (Figure 6A), and quantified the binding capacity against GC-rich sequences as a reference using both GFP activation and BLI assays. The results demonstrated that *Sp*MOON1 bound to AT-rich sequences with an approximately 100-fold higher affinity than to GC-rich sequences (Figure 6B, C). Negative stain EM analysis showed that AT-rich DNA can stabilize and homogenize protein particles, implying the DNA-binding activity (Figure 6D and Supplementary Figure S8A). Further 2D classification results revealed an average particle size of approximately 220 Å (bottom panel of Figure 6D). These data collectively suggest that *Sp*MOON1 is a DNA-binding protein with a preference for AT-rich sequences.

The overall architecture of *Sp*MOON1 includes a forkhead-associated (FHA) domain at the N-terminus, a protein phosphatase 1 (PP1) in the middle, and a repeat region at the C-terminus (Figure 6E). As we synthesized only the TR region of *Sp*MOON1, the DNA-binding ability was not influenced by FHA and PP1 domains. Notably, a similar architecture has been reported for the Ki67 protein in vertebrates, which is a widely used proliferation marker (125). Human Ki67 and *Sp*MOON1 exhibit considerable conservation in their N-terminal regions, both containing FHA and PP1 domains. However, notable differences exist in their C-terminal regions. For instance, human Ki67 encodes 16 repeats of approximately 120 amino acids, including a highly conserved 22 amino acid sequence (TPKEKAQALEDLAGFKELFQTP) known as the Ki67 motif. The Ki67 motif and the repeat unit of *Sp*MOON1 are similar in length but share low identity (Figure 6F and Supplementary Figure S8B), and no evidence has been presented regarding the DNA binding activity of these Ki67 repeats. Interestingly, there is a leucine/arginine-rich (LR) domain at Ki67 C-terminus that has been experimentally demonstrated to bind AT-rich DNA *in vitro* (126). Consequently, we compared the repeat region of *Sp*MOON1 with the LR repeat region of human Ki67, revealing a shared identity of only 22% (Supplementary Figure S8C). Structural comparison was impeded due to the limited confidence in the predicted structure of *Sp*MOON1 (Supplementary Figure S8D and Figure 6G). Taken together, these findings propose a shared N-terminal characteristic between *Sp*MOON1 and the Ki67 protein, while significant variations exist within the DNA-binding activity-conferring region. The conservation of the overall protein architecture and binding preferences provides clues that could help elucidate the potential function of *Sp*MOON1 in *Stylophora pistillata*. Further in-depth research is necessary to elucidate the evolutionary connection between *Sp*MOON1 and human Ki67 repeats.

Next, we retrieved other proteins from the cluster to which *Sp*MOON1 belongs and expanded our search to include unannotated genome data obtained from NCBI, resulting in the identification of a total of 36 homologs, primarily from the *Anthozoa* class (31/36, Supplementary Table S10). The domain architecture of these homologs was primarily defined by an FHA domain at the N-terminus, a PP1 domain in the middle, and varied repeat units at the C-terminus (Figure 6H). Multiple sequence alignment of the repeat units of MOON proteins revealed a high degree of conservation, except for a few positions in the middle (Figure 6H, I). We selected several homologs with varying evolutionary distances and different VAA sets within the hypervariable positions. Specifically, two proteins came from species distantly related to *Stylophora pistillata* (*Acropora digitifera* and *Montipora capitate*), and one was from *Stylophora pistillata*. Accordingly, we named them *Ad*MOON1, *Mc*MOON1, and *Sp*MOON2, respectively. After purifying these proteins, we conducted the BLI-based binding assay, and all three homologs exhibited DNA-binding activity, while only *Sp*MOON2 showed enrichment of a specific motif in the subsequent SELEX (Supplementary Figure S8E and Figure 6J). Notably, the enriched motif displayed an AT-rich pattern similar to that observed in *Sp*MOON1. Further BLI experiments revealed a 10-fold increase in the binding affinity of AT-rich sequences compared to GC-rich sequences, indicating a preference for AT-rich binding (Figure 6K). The similar binding pattern observed between *Sp*MOON1 and *Sp*MOON2 can be attributed to their shared VAA sets, suggesting that the binding preference could be potentially determined by these VAAs.

Collectively, these findings demonstrate broad DNA binding activity within the MOON protein family, with certain members exhibiting a preference for AT-rich sequences.

#### Characterization of pTERF proteins

**Figure 7.**
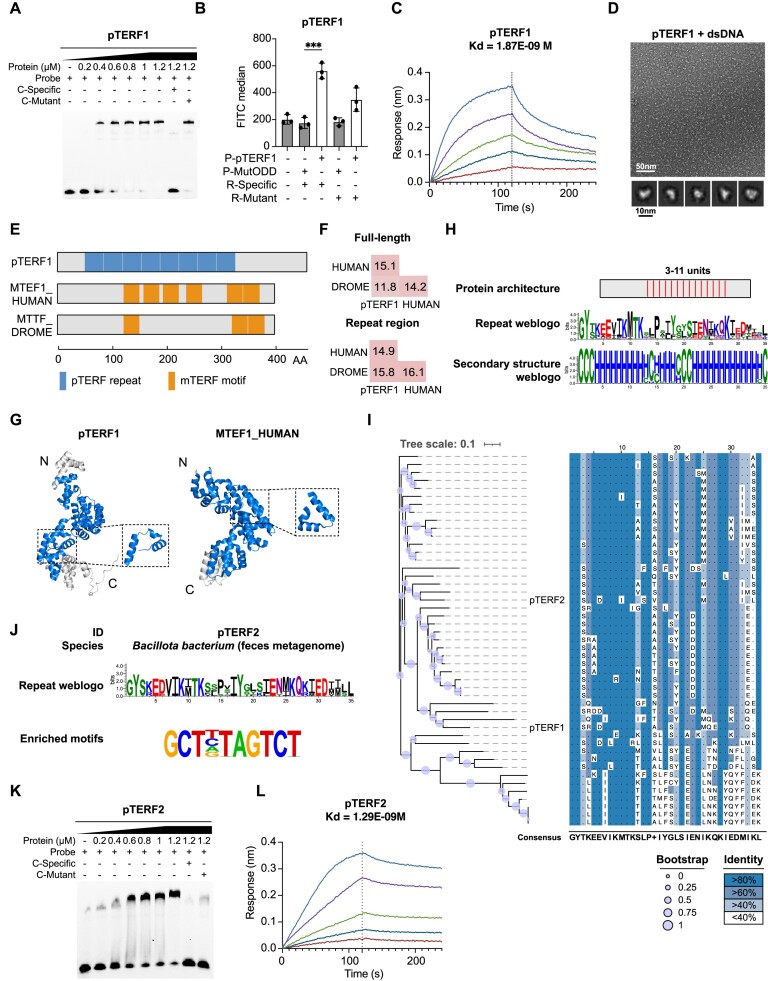
Characterization of the pTERF family. (**A**) EMSA results for pTERF1. The protein concentration was varied, while the probe content was kept constant at 40 nM. (**B**) GFP activation validation for pTERF1. MutODD is a non-DNA binding protein that served as a negative control. ‘P’ and ‘R’ represent protein and reporter plasmids, respectively. Two-sided Student's *t*-test, *n* = 3 biologically independent samples. Data are shown as mean ± s.d. ***P value < 0.001. Data were graphed in GraphPad Prism 8. **C**) BLI results for pTERF1. Protein concentrations were set as follows: 1.48, 2.22, 3.33, 5, 7.5 nM. Data were graphed in GraphPad Prism 8. (**D**) An EM micrograph of negatively stained pTERF1-DNA complex and its representative 2D classification averages. Upper panel scale bar is 50 nm, bottom panel scale bar is 10 nm. (**E**) Protein architecture of the pTERF1, MTEF1_HUMAN and MTTF_DROME proteins. The architecture of MTEF1_HUMAN and MTTF_DROME was derived from the literature (129). (**F**) Sequence comparison of pTERF1, MTEF1_HUMAN and MTTF_DROME. (**G**) Protein tertiary structure of pTERF1 and MTEF1_HUMAN (PDB: 3MVA). Structures of pTERF1 were predicted by AlphaFold2. The expanded box illustrates the structure of a single repeat unit or an mTERF motif. (**H**) Domain architecture, repeat and secondary structure weblogos of the pTERF family. Red rectangles represent the repeat units within pTERF proteins, ranging from 3 to 11. (**I**) Multiple sequence alignment and phylogenetic tree of the pTERF family. The names of experimentally tested homologs of pTERF1 are labeled. In each aligned column, the degree of conservation is represented by the undertone of the amino acid. The bootstrap confidence scores are represented by circle sizes. The color and identity coordinates are shown on the right. (**J**) Species, repeat logos and enriched motifs of pTERF2. (**K**) EMSA results for pTERF2. The protein concentration was varied, while the probe content was kept constant at 40 nM. (**L**) BLI results for pTERF2. Protein concentrations were set as follows: 0.625, 1.25, 2.5, 5, 10 nM. Data were graphed in GraphPad Prism 8.

The other DNA-binding TRP identified via the *in vitro* screening was from the ruminant gut metagenome, which binds the motif ACTNNNAGTC (Figure 3C). The resource metagenomic-assembled genome was taxonomically classified as *Clostridia bacterium*, an unclassified bacterium within the *Bacillota* phylum (127). We first confirmed the binding activity of this TRP by EMSA and GFP activation assays (Figure 7A, B). BLI assays were further performed to quantify the binding affinity, revealing a Kd value of 1.87 nM (Figure 7C). Moreover, we observed a relatively uniform distribution of protein particles in the presence of DNA (Figure 7D and Supplementary Figure S9A). Further 2D classification results revealed an average particle size of approximately 90 Å (bottom panel of Figure 7D).

Notably, several mTERF (mitochondrial transcription termination factor) motifs were annotated within the repeat region. We have accordingly named it as **p**rokaryotic m**TERF**-like protein, specifically designated as pTERF homolog 1 (pTERF1). MTERF motifs are non-tandem repeats and have been well documented as encoding functional nucleic acid-binding proteins in eukaryotes (128). However, currently, there is no evidence suggesting the existence of a prokaryotic homolog for the mTERF motif. Thus, we conducted a comparative analysis between pTERF1 and human mTERF1 (UniProt entry name: MTERF1_HUMAN) and Drosophila DmTTF (UniProt entry name: MTTF_DROME) (129). The repeats within the MTERF1_HUMAN and MTTF_DROME proteins are interspersed throughout the protein sequence, while the repeats in pTERF1 are arranged in a tandem manner (Figure 7E). Remarkable sequence variability was evident among these three proteins (Figure 7F), consistent with a previous study (129). Although MTERF1_HUMAN and MTTF_DROME shared low identity of their sequences, they retained conserved mTERF motif characteristics, including the preservation of a proline at position 8, along with a leucine or another hydrophobic amino acid at positions 11, 18, and 25, forming three leucine zipper (LZ)-like heptads X_3_LX_3_ (129). Notably, these conserved features were not observed in pTERF1 (Figure 3C). Given the absence of obvious sequence similarity, we conducted a structural-level comparative analysis between pTERF1 and MTERF1_HUMAN (PDB accession: 3MVA). The repeat region of pTERF1 showed well-ordered structure with an average pLDDT score exceeding 80 (Supplementary Figure S9B), and the general structures of the pTERF1 repeat unit and the single mTERF motif were similar, with each being composed of three α-helices. Nevertheless, differences were apparent, including variations in the number of helical turns and the local conformation of the individual unit (Figure 7G).

To elucidate the evolutionary relationship between pTERF1 and the eukaryotic mTERF family, we conducted a comparative analysis encompassing all experimentally characterized mTERF homologs documented within the UniProtKB/Swiss-Prot database. A total of 30 mTERF protein sequences and their corresponding tertiary structures were retrieved for investigation. Among them, the tertiary structures of two human mTERF proteins, MTERF1_HUMAN and MTERF3_HUMAN, were extracted from the PDB database, while the rest were obtained from the Alphafold2 database. As demonstrated in Supplementary Figure S9C, a sequence-level comparison revealed the presence of several mTERF subtypes, including mTERF1, mTERF2, mTERF3 and mTERF4. Notably, plant mTERF4 and mammalian mTERF4 were clustered in different groups, revealing distinct evolutionary paths. Notably, pTERF1 displayed limited sequence identity with all eukaryotic-origin mTERF proteins, ranging from 9% to 18%. This observation potentially implies a unique evolutionary trajectory of pTERF1 compared with eukaryotic mTERFs. In contrast to sequence-level comparisons, structural analysis, as depicted by the root mean square deviation (RMSD), revealed a relatively conserved relationship among distinct subtypes (Supplementary Figure S9D). These findings support the existence of a certain level of functional conservation among these proteins despite limited sequence similarity. Importantly, both mammalian and plant mTERFs are nucleus-encoded, although they function in mitochondria or chloroplasts. Emerging evidence supports the idea that bacterial genes contribute to the origination and evolution of mitochondria (130,131). Hence, the identification of pTERF1 raises the exciting possibility of the presence of the first prokaryotic-origin mTERF-like protein.

To further characterize the pTERF family, we investigated the homologs of pTERF1 within the same cluster and recovered five proteins. Deep homolog searching in the NCBI database identified 14 additional homologs (Supplementary Table S11). Most of them had a unit number below six and therefore were not included in the original list of TRs of interest (Figure 7H). All of these proteins were obtained from the metagenomic-assembled genomes (MAGs). The MAG assembly completeness levels ranged from approximately 70% to 100%, and contamination levels from 0% to 10%, indicating a medium to high quality of the assembly. Additionally, all the MAGs were taxonomically classified within the *Bacteria* kingdom, with the majority falling within the *Bacillota* phylum. In order to confirm the widespread presence of pTERFs in metagenomic ecosystems, we further conducted a search for homologous sequences in the MGnify database, and successfully identifying an additional 27 homologs (132) (Supplementary Table S11). To investigate whether other proteins within the pTERF family also possessed DNA binding ability, we selected several genes for analysis from various positions on the phylogenetic tree, while only one gene (pTERF2) was successfully synthesized (Figure 7I). The protein was purified, and a subsequent SELEX was conducted. Interestingly, a motif different from that of pTERF1 was enriched (Figure 7J). We further validated DNA binding activity by EMSAs and BLI assays (Figure 7K, L). The obtained data indicated that distinct repeat arrangements of pTERF1 and pTERF2 resulted in different binding specificities, suggesting potential reprogrammability.

To gain initial insights into the functions of pTERF proteins, we conducted an analysis to predict the potential gene functions targeted by pTERF1 and pTERF2. As indicated in Supplementary Figure S9E, F, pTERF1 and pTERF2 were associated with similar GO terms related to RNA metabolic processes. Collectively, these data revealed the binding characteristics of pTERF family, and highlight the discovery of the first class of distant mTERF homologs in prokaryotes, shedding light on their evolutionary significance and functional diversity. Furthermore, the recognition of diverse DNA sequences is facilitated by the different arrangements of repeats within the protein family, suggesting the intriguing possibility of reprogrammability.

## Discussion

TRPs are widely distributed across the tree of life. However, only a handful of TRP families have been identified and studied in depth (31–33) (Supplementary Table S1). The investigation of these TRPs yielded significant biological discoveries, and the mechanistic understanding of some led to the development of novel technologies, such as ZFN and TALEN (66,133). In this study, we conducted a comprehensive analysis of the TRPs in the current protein database, and found that the majority of them are unexplored. As an initial step towards uncovering the diversity of these TRPs, we identified 11 new DNA-binding TRPs, among which six showed DNA sequence recognition specificity.

Advances in computational tools, especially deep learning tools, greatly facilitated our efforts, primarily in two regards: (i) We harnessed large language models to predict DBPs. By integrating three large PLMs, employing additional neural network layers, and curating a training and testing dataset with improved quantity and quality, we established the PLM-DBPPred model that achieved state-of-the-art performance in predicting DBPs. Using this tool, we predicted 8,865 novel DNA-binding TRPs. (ii) We employed AlphaFold2 to predict the tertiary structure of candidate proteins. Interestingly, we achieved good quality structure prediction for proteins (*Pq*STAR1, *Asp*STAR1 and pTERF1) that are distantly related to or share similar tertiary structure features with known proteins (AvrBs3 and human mTERF1). This is consistent with the fact that Alphafold2 exploits evolutionary information inferred from the multiple sequence alignment of homologous proteins (22,102). In contrast, structural prediction for three proteins (*Sp*MOON1, *Tc*NORD1 and *Aa*MIRAGE1) was poor, likely due to different reasons. Specifically, *Tc*NORD1 and *Aa*MIRAGE1 exhibited high disordered scores spanning the repeat region, preventing attainment of a stable three-dimensional native structure. Previous studies have suggested that certain proteins are disordered in isolation but can adopt a folded structure upon ligand binding (134,135). Furthermore, there is a notable prevalence of intrinsic disorder observed in transcription factors, with AT-hooks serving as a prominent example (136). Further efforts, such as structure resolution, are necessary to determine whether these two disorder proteins we have identified exhibit a similar behavior. In the case of *Sp*MOON1, the challenge may stem from its lack of previously studied proteins that are remotely similar. This highlights the inherent constraint of deep learning approaches, which rely on existing databases and knowledge. Systematic functional screening has the potential to unveil novel protein types that exceed the predictive capabilities of these models and fill knowledge gaps.

The identification of *Tc*NORD1 suggests intriguing evidence of DNA binding activity in neuropeptide-like protein. Analysis of MIRAGE family revealed integration of DNA-binding repeat modules within multifunctional DBHS-like protein, potentially expanding the structural modularity and functional diversity. The MOON family was identified as a novel type of DBP in marine organisms, with the possibility of functioning analogously to the human Ki67 protein. Additionally, the identification of pTERF proteins indicates the potential prokaryotic homologs of mTERF proteins. In addition, *Pq*STAR1, characterized by a small number of repeat units, demonstrates a higher DNA-binding affinity compared to canonical TALEs. *Pq*STAR1 can be readily reprogrammed to target specific DNA sequences with high activity, and the T0 preference of canonical TALEs was not observed. Since most eukaryotic TF binding motifs are around 10 bps (137), *Pq*STARs are uniquely suited for generating ATFs that mimic natural TFs, as their target site length is as short as 9 bp. Utilizing these distinctive features of *Pq*STAR, we developed STAR-based ATFs, which enhanced the expression of a large number of endogenous genes with shared regulatory motif. Previous studies have described the application of ATFs or TF libraries by fusing gene regulatory domains to ZNFs, TALEs, or nuclease deactivated Cas9 (dCas9) (107,108,138). However, the inherent features of these systems impose constraints on modeling natural TFs, such as the ‘neighboring effect’ of ZNFs (139), insufficient short target (<10 bp) binding capacity of canonical TALE and dCas9 (104,140). Therefore, *Pq*STAR provides a unique platform for constructing ATFs that regulate transcription network. Systematically evaluating the binding affinity and specificity of varying numbers of repeats would further elucidate the potential range of function optima (104). We also identified *Asp*STAR1, the first TALE-like protein in eukaryotes. Further study will elucidate its evolutionary origins and biological functions.

Besides leveraging TRP to develop gene editing and regulatory tools, certain types of TRs exhibit lineage-specific conservation and interspecies variability, making them useful markers for genotyping (141). Our preliminary protein-level TR analysis indicated that approximately half of the TR clusters are species-specific (Supplementary Figure S1F). Further analysis of these lineage-specific TRs is expected to identify markers for genotyping, enhancing our understanding of species evolution and providing potential valuable markers for precise genetic identification.

Despite employing state-of-the-art DBP prediction and implementing both *in vitro* and *in vivo* functional screening strategies, only 11 DBPs out of 100 candidates were identified as DNA-binding. This suggests limitations of both the computational analysis and functional screening in our current study. On the computational side, a TR-based DBP predictive model would be more relevant. We tried to build a classification model using ZNFs and TALEs (the two major classes of TR-DBPs currently known) as the positive training set and a randomly sampled negative training set from UniProt. However, the model demonstrated poor performance (data not shown), indicating that insufficient diversity impaired model generalization capability. Our current study, surely far from perfect, offered a starting point for expanding the diversity of TR-DBPs. Another very important factor that will improve our analysis and prediction is to incorporate protein structure information predicted by tools such as AlphaFold2 (102). The absence of the incorporation of predicted structural information in our upstream analysis pipeline is due to the fact that the project started five years ago when AlphaFold2 had not yet been launched. Further incorporation of structure information predicted by AlphaFold3 (142) promises to greatly enhance the efficiency of identifying novel TR-DBPs. On the functional screening side, incorporating technologies such as high-throughput *in vitro* translation has the potential to significantly improve our platform (143,144). Moreover, applying more diverse functional assays, such as large-scale protein arrays or multiplex SELEX analyses, could further enhance the throughput, leading to the identification of a greater number of positive hits (98,145–149).

In summary, our work established a platform combining deep learning-driven bioinformatics and a standardized experimental pipeline for the systematic discovery of DNA-binding TRPs, based on which multiple novel families with unique features were identified. By investigating a small representative set of TR families, we provided a starting point and reference for researchers interested in mining TR in a larger scale. By modulating the analysis parameters and corresponding experimental setup, proteins with certain defined features and functions, such as RNA-binding proteins, could be systematically explored in a similar manner. We believe that this data-driven approach will accelerate the discovery of new biological insights and biotechnologies, and our efforts are only a small step in this direction.

## Supplementary Material

gkae710_Supplemental_Files

## Data Availability

This paper analyzed existing, publicly available data. The accession numbers for the datasets are listed in the Supplementary Tables. Source codes for PLM-DBPPred are available at https://zenodo.org/doi/10.5281/zenodo.10675351. The in-house scripts and codes for analyzing TRPs are available at https://doi.org/10.5281/zenodo.10823513. The raw next-generation sequencing data for CUT&Tag, RNA-seq, SELEX, and B1H have been deposited in the Genome Sequence Archive of the Beijing Institute of Genomics, Chinese Academy of Sciences, and can be accessed through the following links: https://bigd.big.ac.cn/gsa-human/browse/HRA006860 for CUT&Tag and RNA-seq and https://bigd.big.ac.cn/gsa/browse/CRA015384 for SELEX and B1H. Any additional information required to reanalyze the data reported in this paper is available from the lead contact upon request.
