# Supplementary material for: Systematic discovery of DNA-binding tandem repeat proteins
Source: Nucleic Acids Res. 2024 Aug 27;52(17):10464–89. doi: 10.1093/nar/gkae710 (PMC11417379; doi:10.1093/nar/gkae710)
Supplement: gkae710_Supplemental_Files [file gkae710_supplemental_files.zip › Supplementary information.pdf]

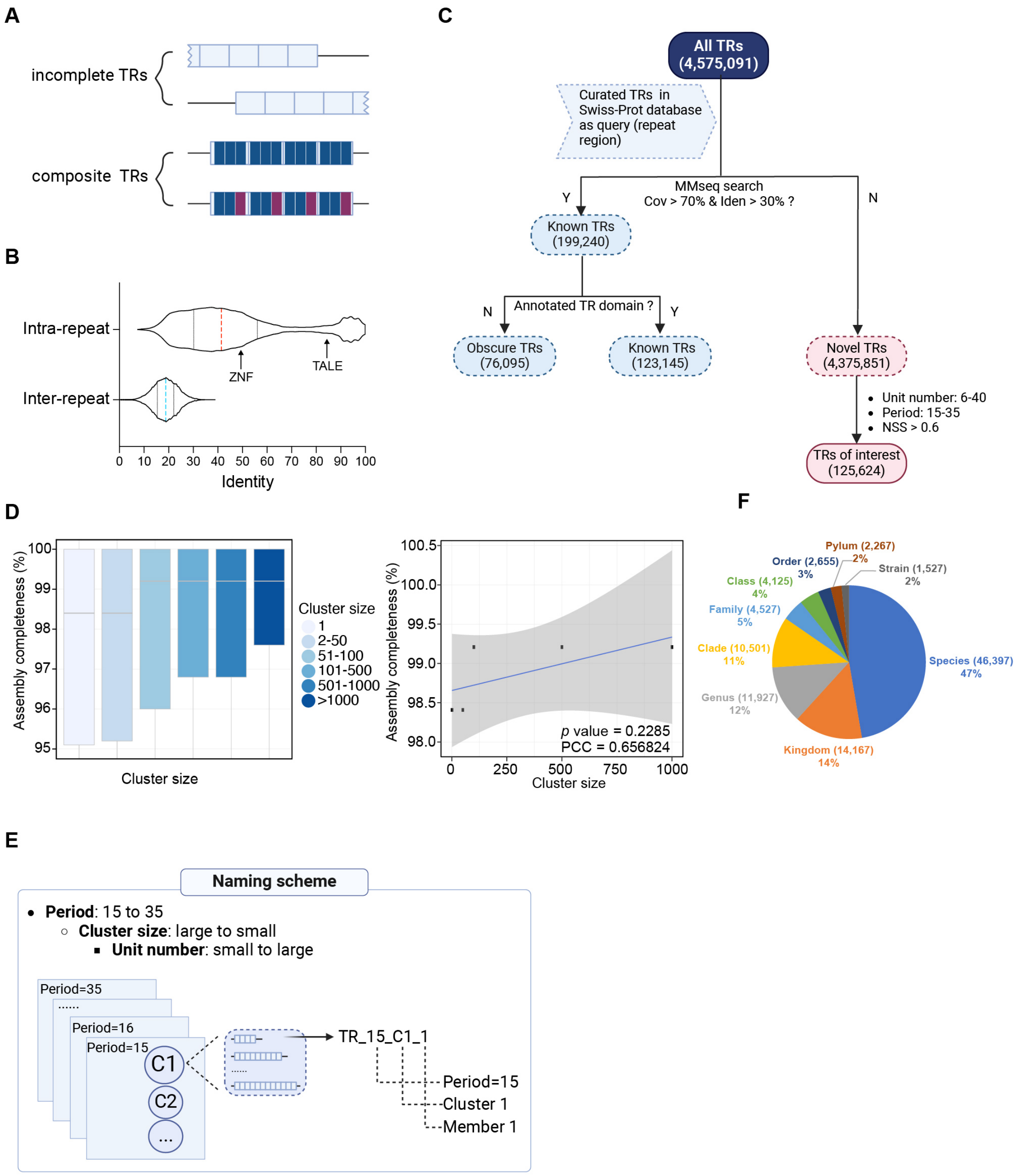

Supplementary Figure 1. Detailed workflow for tandem repeat proteins analysis. **A)** Schematic illustrating the “incomplete TR” and “composite repeat”. **B)** Distribution of identity scores for intra-repeat and inter-repeat comparisons. The prefix “intra-” denotes comparisons within a repeat family, while the prefix “inter-” denotes comparisons between ZNF and TALE repeats. **C)** Detailed workflow for identifying TRs. **D)** The upper panel illustrates the distribution of cluster size and the completeness of genome assemblies. Specifically, for TRs within each cluster size range, we retrieved the corresponding genome assemblies and then randomly selected 2,000 of them for assessing genome completeness. The lower panel represent the Pearson correlation analysis between cluster size and the median statistics for genome assembly completeness. **E)** Diagram illustrating the naming rules for TRs. **F)** Distribution of TR

clusters confined to each taxonomic rank attending to the last common ancestor (LCA) of their members.

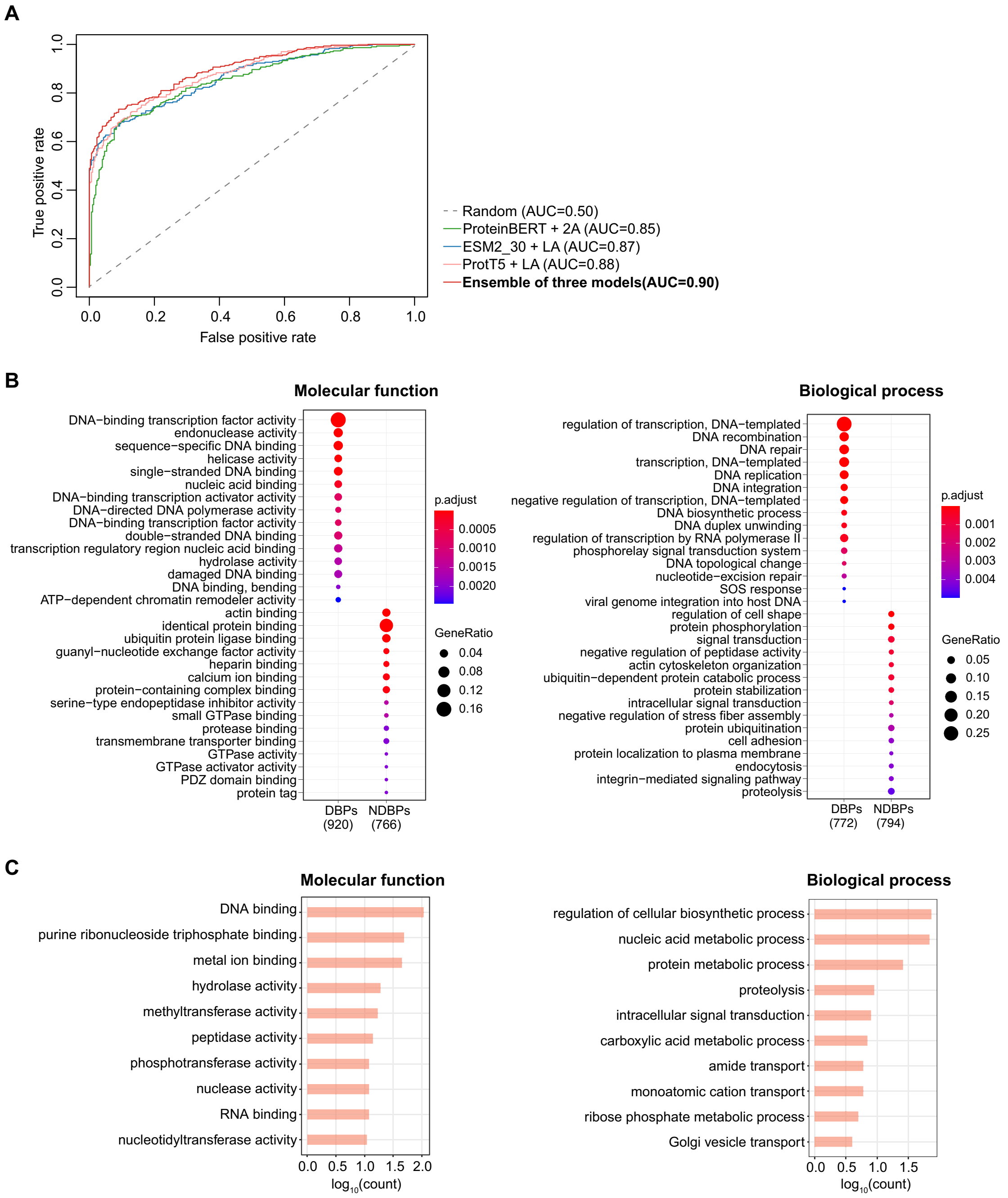

Supplementary Figure 2. Model performance evaluation for identifying DBPs and subsequent functional analysis.

A) ROC-AUC analysis evaluates the performance of various model combinations. Area under curve (AUC) values are indicated for each classifier. 2A: two-layer attention; LA: light attention. **B)** GO Enrichment analysis for DBPs and NDBPs in PDB database. **C)** GO Enrichment analysis for DNA binding domain (DBD) partner domain.

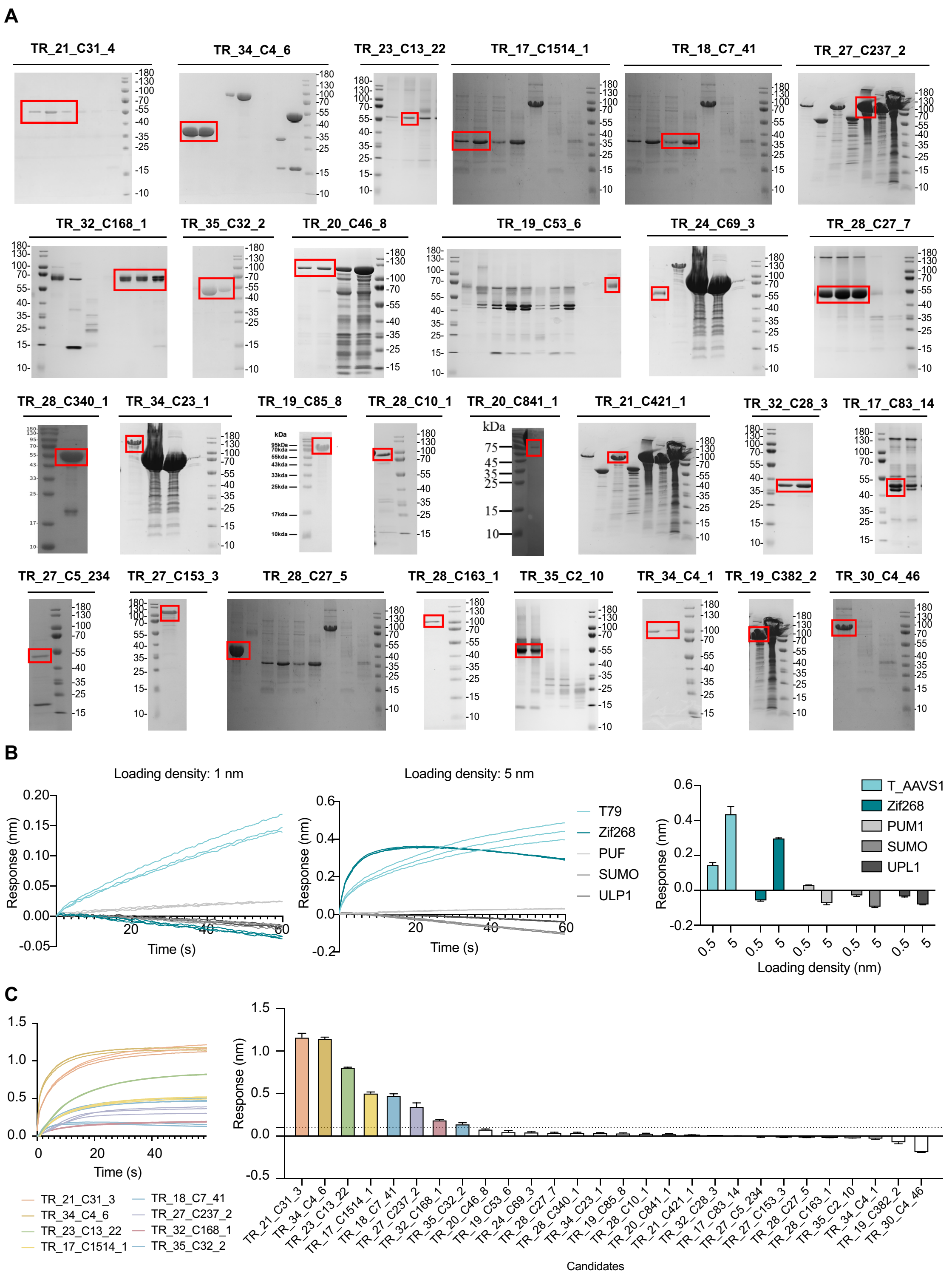

Supplementary Figure 3. The SDS-PAGE analysis and results for BLI-based screening.

A) SDS-PAGE gels for 28 purified proteins. **B)** Feasibility test for the BLI-based screening assay. The line plot represents BLI traces acquired with immobilized proteins on Ni-NTA biosensors. The bar plot represents binding response for each protein. Three technical replicates were conducted for each protein. Zif268 and T_AAVS1 were included as positive controls, while PUM1, SUMO, and ULP1 were designated as

negative controls. Two protein loading densities, 1 nm and 5 nm, were assessed. **C)** Results of the BLI-based screening assay. The line plot represents BLI traces acquired with immobilized proteins on Ni-NTA biosensors. The bar plot represents binding response for each protein. Three technical replicates were conducted for each protein. The red dash line indicates the binding response cutoff. Candidate proteins with potential binding activity are indicated by colored solid columns, while proteins without potential binding activity are depicted as hollow columns.

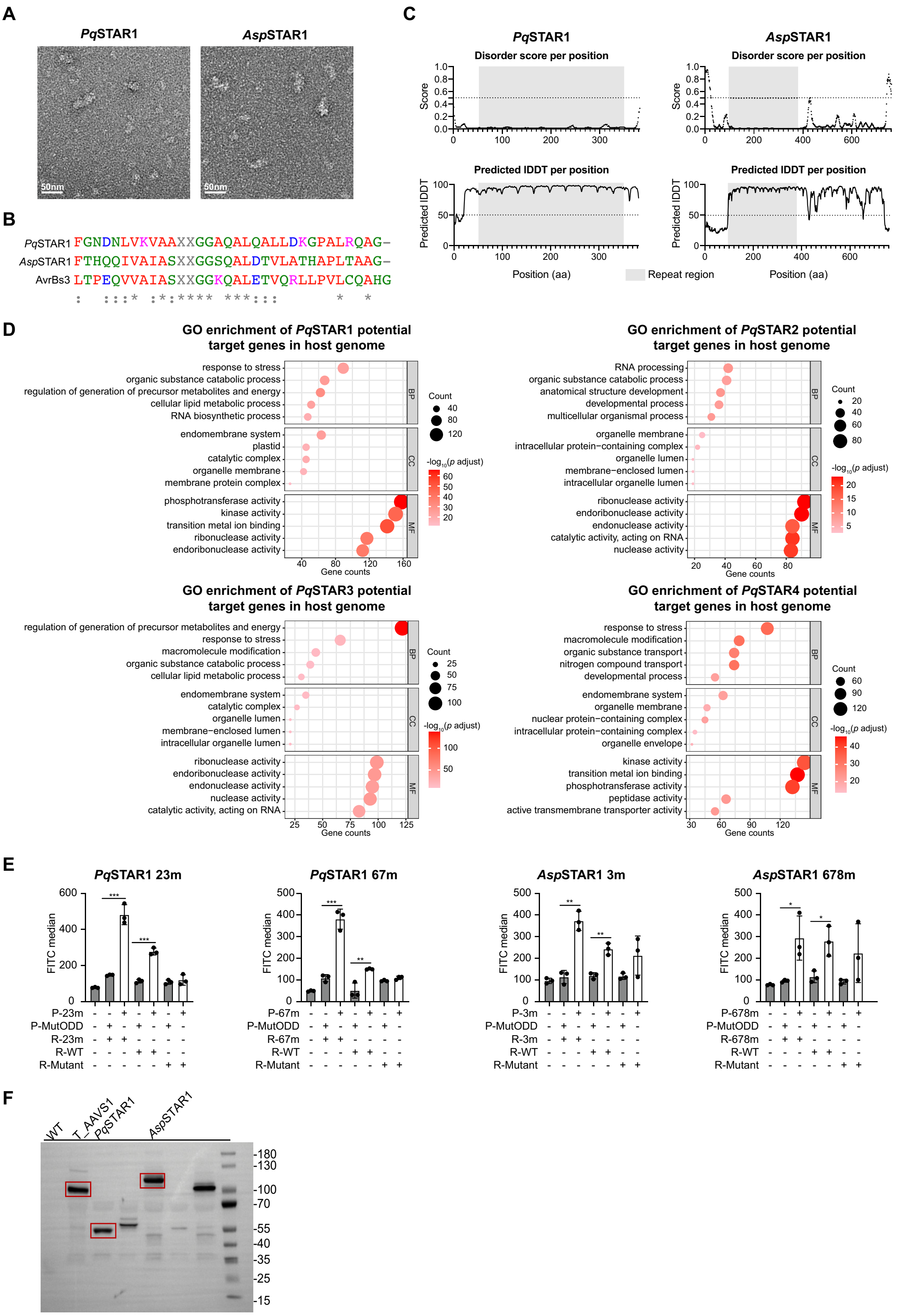

Supplementary Figure 4. Detailed characterization of STAR family.

A) EM micrographs of negatively stained *Pq*STAR1 and *Asp*STAR1. Scale bar, 50 nm. **B)** Multiple sequence alignment of repeat consensus sequences for *Pq*STAR1, *Asp*STAR1 and AvrBs3. Residues are colored according to the Clustal omega color

code: AVFPMILW, red (small + hydrophobic); DE, blue (acidic); RHK, magenta (basic); STYHCNGQ, green (Hydroxly + sulfhydryl + amine + G); others, gray (unusual amino acid). The asterisk (*) and colon (:) indicate identical amino acid residues and conserved substitutions in all sequences used in the alignment, respectively. **C)** Predicted disorder score (upper panel) and predicted local distance difference test (LDDT) score (bottom panel) per position for *Pq*STAR1 and *Asp*STAR1. Data were graphed in GraphPad Prism 8. **D)** GO enrichment of potential target genes regulated by *Pq*STAR1, *Pq*STAR2, *Pq*STAR3 and *Pq*STAR4 in host genome. The top 5 enriched terms for each category were shown. “BP”, “CC” and “MF” represent biological process, cellular component and molecular function, respectively. **E)** GFP activation validation for *Pq*STAR1 and *Asp*STAR1 variants. For *Pq*STAR1, WT, 23m, and 67m represent the wild-type protein, a variant with modified RVD2 and RVD3, and a variant with modified RVD6 and RVD7, respectively. For *Asp*STAR1, WT, 3m, and 678m represent the wild-type protein, a variant with modified RVD3, and a variant with modified RVD6, RVD7 and RVD8, respectively. MutODD is a non-DNA binding protein that served as a negative control. “P” and “R” represent protein and reporter plasmid, respectively. Two-sided Student’s t-test, $n = 3$ biologically independent samples. Data are shown as mean ± s.d. *, *p value* < 0.05; **, *p value* < 0.01; ***, *p value* < 0.001. Data were graphed in GraphPad Prism 8. **F)** Western blot results of *Pq*STAR1 and *Asp*STAR1expression in 293T cells.

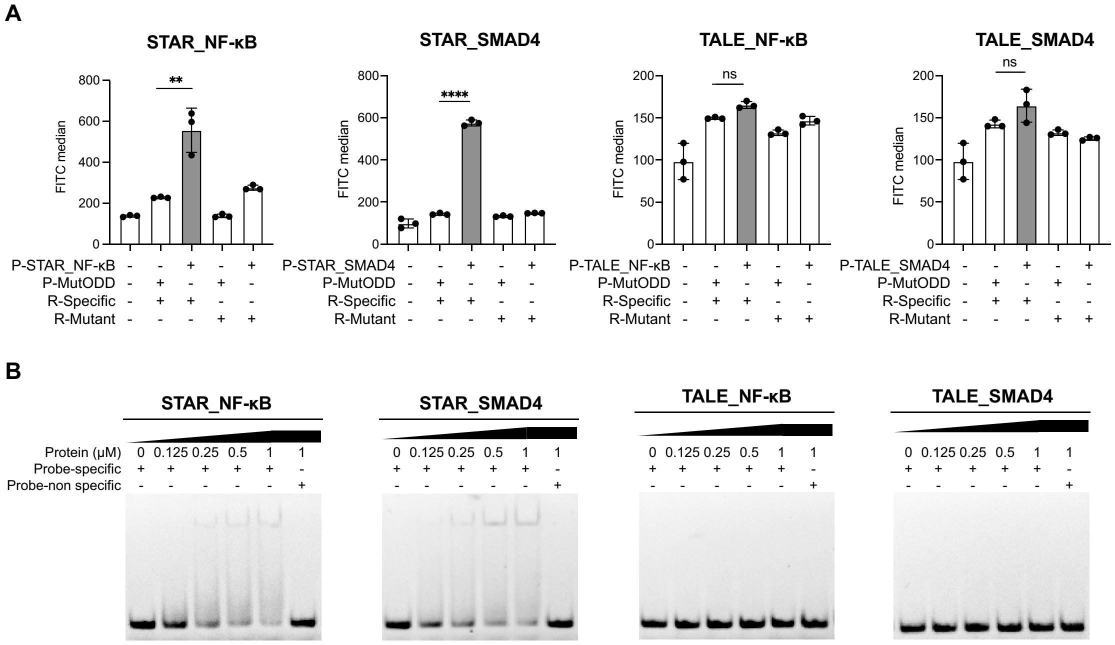

Supplementary Figure 5. Comparison between artificial STARs and TALEs.

A) GFP activation results for artificial STARs/TALEs targeting NF-κB and SMAD4 binding motifs. MutODD is a non-DNA binding protein that served as a negative control. "P" and "R" represent protein and reporter plasmid, respectively. Two-sided Student's t-test, n = 3 biologically independent samples. Data are shown as mean ± s.d. *, *p value* < 0.05; **, *p value* < 0.01; ***, *p value* < 0.001, ns, not significant. Data were graphed in GraphPad Prism 8. **B)** EMSA results for artificial STARs/TALEs targeting NF-κB and SMAD4 binding motifs. The protein concentration was varied, while the probe content was kept constant at 40 nM.

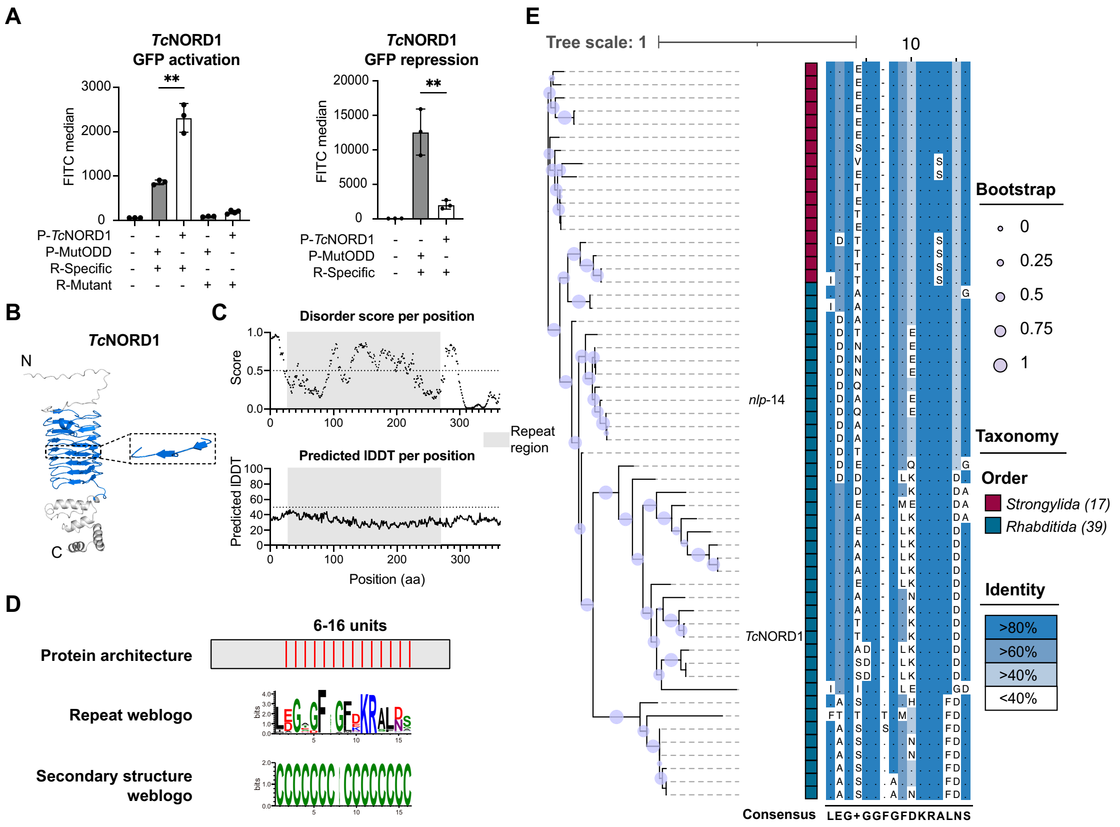

Supplementary Figure 6. Characterization of NORD family.

A) The GFP activation and repression validation results for *Tc*NORD1. MutODD is a non-DNA binding protein that served as a negative control. "P" and "R" represent protein and reporter plasmid, respectively. Two-sided Student's t-test, n = 3 biologically independent samples. Data are shown as mean ± s.d. **, *p value* < 0.01. Data were graphed in GraphPad Prism 8. **B)** Predicted protein tertiary structure for *Tc*NORD1. The expanded box highlights the structure of a single repeat unit. N and C denote the N-terminal and C-terminal ends, respectively. **C)** Predicted disorder score (upper panel) and predicted local distance difference test (LDDT) score (bottom panel) per position for *Tc*NORD1. Data were graphed in GraphPad Prism 8. **D)** Domain architecture, repeat and secondary structure weblogos of NORD repeat family. Red rectangles present the repeat units within NORD repeat family, ranging from 6 to 16. **E)** Multiple sequence alignment and phylogenetic tree of the proteins in NORD. The phylogenetic tree was constructed using repeat region sequences. In each aligned column, the degree of conservation is represented by the undertone of the amino acid. The bootstrap confidence scores are represented by circle sizes. Squares with different colors in the middle represent different taxa. The color and identity coordinates are shown on the right.

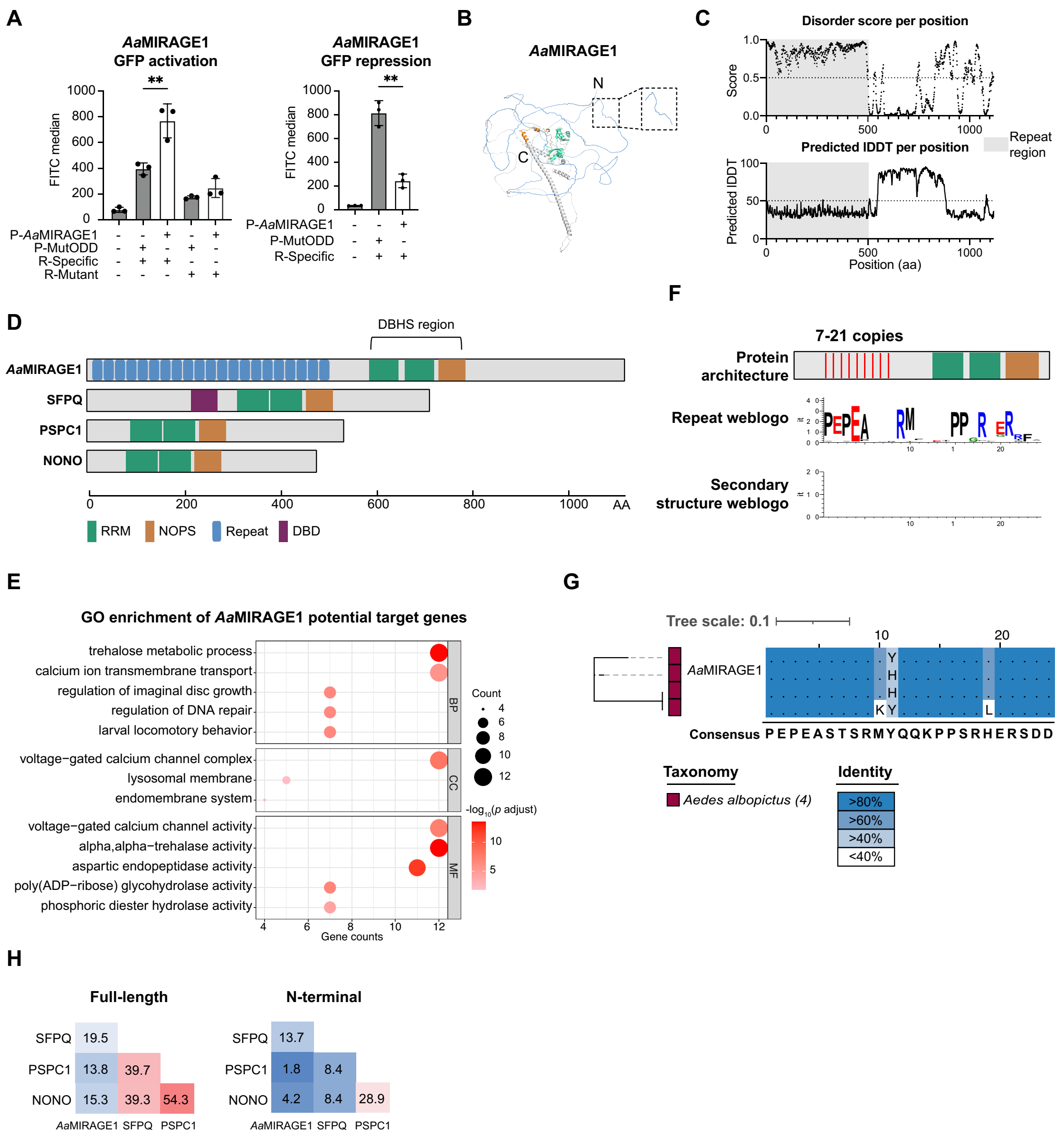

Supplementary Figure 7. Characterization of MIRAGE family.

A) The GFP activation and repression validation results for *Aa*MIRAGE1. MutODD is a non-DNA binding protein that served as a negative control. "P" and "R" represent protein and reporter plasmid, respectively. Two-sided Student's t-test, n = 3 biologically independent samples. Data are shown as mean ± s.d. **, *p value* < 0.01. Data were graphed in GraphPad Prism 8. **B)** Predicted protein tertiary structure for *Aa*MIRAGE1. The RRM, NOPS domain and repeat region are colored green, orange and blue, respectively. The expanded box highlights the structure of a single repeat unit. **C)** Predicted disorder score (upper panel) and predicted local distance difference test (LDDT) score (bottom panel) per position for *Aa*MIRAGE1. Data were graphed in GraphPad Prism 8. **D)** Protein architecture of the *Aa*MIRAGE1, SFPQ, PSPC1 and NONO proteins. The architecture of SFPQ, PSPC1 and NONO was derived from the literature[1]. **E)** GO enrichment of potential target genes regulated by *Aa*MIRAGE1. The top 5 enriched terms for each category were shown. "BP", "CC" and "MF" represent

biological process, cellular component and molecular function, respectively. **F)** Domain architecture, repeat and secondary structure weblogos of MIRAGE repeat family. Red rectangles present the repeat units within MIRAGE repeat family, ranging from 7 to 21. **G)** Multiple sequence alignment and phylogenetic tree of the proteins in MIRAGE. The phylogenetic tree was constructed using repeat region sequences. In each aligned column, the degree of conservation is represented by the undertone of the amino acid. The bootstrap confidence scores are represented by circle sizes. Squares with different colors in the middle represent different taxa. The color and identity coordinates are shown on the right. **H)** Sequence comparison of *Aa*MIRAGE1, SFPQ, PSPC1 and NONO proteins.

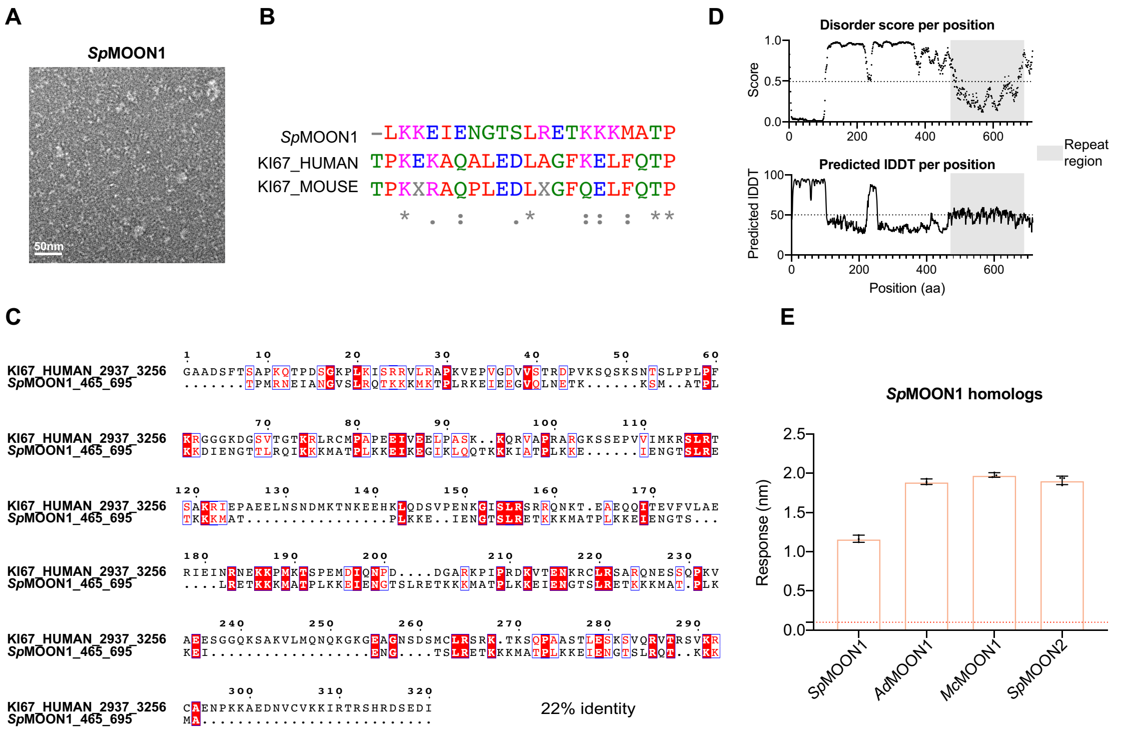

Supplementary Figure 8. Detailed characterization of MOON family.
A) An EM micrograph of negatively stained *Sp*MOON1. Scale bar, 50 nm. **B)** Multiple sequence alignment of repeat consensus sequences for *Sp*MOON1, KI67_HUMAN and KI67_MOUSE protein. Residues are colored according to the Clustal omega color code: AVFPMILW, red (small + hydrophobic); DE, blue (acidic); RHK, magenta (basic); STYHCNGQ, green (Hydroxly + sulfhydryl + amine + G); others, gray (unusual amino acid). The asterisk (*), colon (:), and dot (.) indicate identical amino acid residues, conserved substitution, and semi-conserved substitutions in all sequences used in the alignment respectively. **C)** Multiple sequence alignment of LR domain within KI67_HUMAN protein and repeat region within *Sp*MOON1. Red box, white character represents strictly conserved residues. Red character with white box represents similar residues. **D)** Predicted disorder score (upper panel) and predicted local distance difference test (LDDT) score (bottom panel) per position for *Sp*MOON1. Data were graphed in GraphPad Prism 8. **E)** BLI screen results for *Sp*MOON1 homologs. Data were graphed in GraphPad Prism 8.

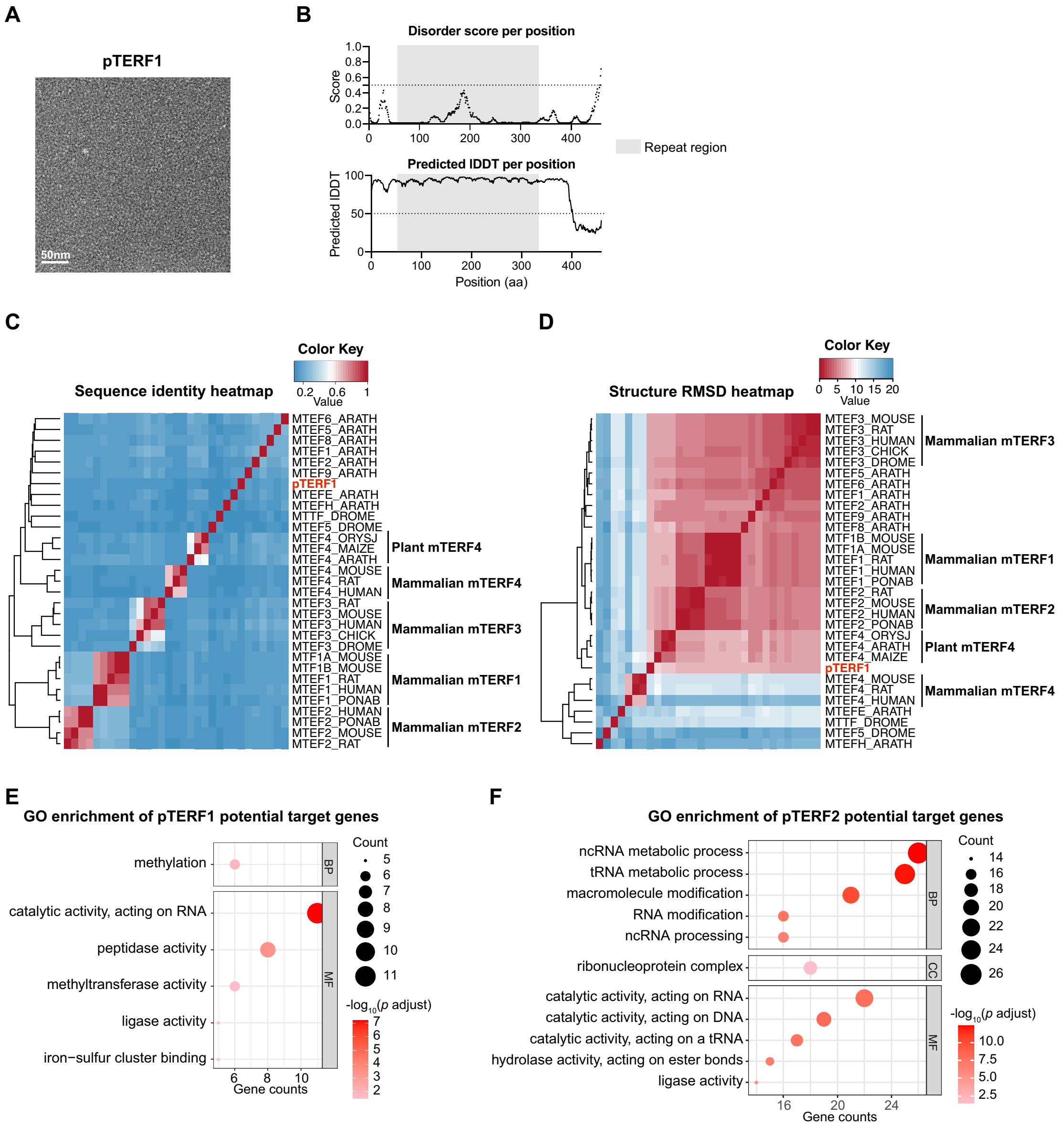

Supplementary Figure 9. Detailed characterization of pTERF family.

A) An EM micrograph of negatively stained pTERF1. Scale bar, 50 nm. **B)** Predicted disorder score (upper panel) and predicted local distance difference test (LDDT) score (bottom panel) per position for pTERF1. Data were graphed in GraphPad Prism 8. **C)** Sequence identity heatmap of pTERF and eukaryotic mTERF proteins. Protein accession is corresponding to the entry name in UniProt database. HUMAN, ARATH, DROME, ORYSJ, MAIZE, MOUSE, RAT, PONAB represent Homo sapiens, Arabidopsis thaliana, Drosophila melanogaster, Oryza sativa, Zea mays, Mus musculus, Rattus norvegicus, Pongo abelii, respectively. **D)** Structure RMSD heatmap of pTERF and eukaryotic mTERF proteins. Smaller values of RMSD correspond to higher structural similarity. **E)** GO enrichment of potential target genes regulated by pTERF1. The top 5 enriched terms for each category were shown. "BP", "CC" and "MF" represent biological process, cellular component and molecular function, respectively. **F)** GO enrichment of potential target genes regulated by pTERF2.

Supplementary Table 1: Pfam accessions of well-studied TR families from literatures and databases.

Supplementary Table 2: Protein information for "TRs of interest".

Supplementary Table 3: Information of existing DBP prediction tools.

Supplementary Table 4: Evaluation results and hyperparameters of DBP prediction models.

Supplementary Table 5: Pfam accessions of DNA-related domains.

Supplementary Table 6: Protein information of synthesized candidates.

Supplementary Table 7: Protein information of STAR family.

Supplementary Table 8: Protein information of NORD family.

Supplementary Table 9: Protein information of MIRAGE family.

Supplementary Table 10: Protein information of MOON family.

Supplementary Table 11: Protein information of pTERF family.

References

1 Knott, G. J., Bond, C. S. & Fox, A. H. The DBHS proteins SFPQ, NONO and PSPC1: a multipurpose molecular scaffold. *Nucleic acids research* **44**, 3989-4004 (2016).
